# ActivityFinder: Toward the Fully Automatic Integration of Structural and Binding Affinity Data

**DOI:** 10.1021/acs.jcim.5c02505

**Published:** 2026-01-02

**Authors:** Emanuel S. R. Ehmki, Torben Gutermuth, Tobias Harren, Stefan Kurtz, Matthias Rarey

**Affiliations:** ZBH - Center for Bioinformatics, 14915 University of Hamburg, Albert Einstein Ring 8-10, 22761 Hamburg, Germany

## Abstract

The reliable integration of structural and bioactivity data remains a significant bottleneck in computational chemistry and cheminformatics. While curated databases such as PDBbind and ChEMBL provide valuable resources, integrating structural data in the form of, for example, PDB files and bioactivity assays in the form of structured data, like ChEMBL, is inherently complex, and no fully integrated solution has been published so far. This work introduces ActivityFinder, a fully automated method for linking protein–ligand crystal structures to bioactivity assay data without relying on external services or continuous data connections. The method solely requires structural information in the form of PDB files and a structured SQL database such as ChEMBL, making it highly suitable for proprietary or unpublished data sets typically used within the pharmaceutical industry or early research in general. ActivityFinder utilizes sequence alignments and detailed chemical structure matching. Its accuracy is showcased for the task of associating PDB entries with corresponding data in ChEMBL. Applying this method, we linked 20197 PDB structures and 13734 ligands with 17829 unique ChEMBL ligands across 2585 targets, covering over one million bioactivity data points. Compared to existing approaches based on identifier mapping, ActivityFinder reproduces reported links but also broadens the set of linked data by explicitly addressing ligand heterogeneity, sequence variants, and binding-site mutations at an atomistic level. ActivityFinder is available via the Rest API of the ProteinsPlus platform, and the data is published as a PostgreSQL database dump, enabling scientists to integrate and explore structural and bioactivity data reliably.

## Graphical abstract

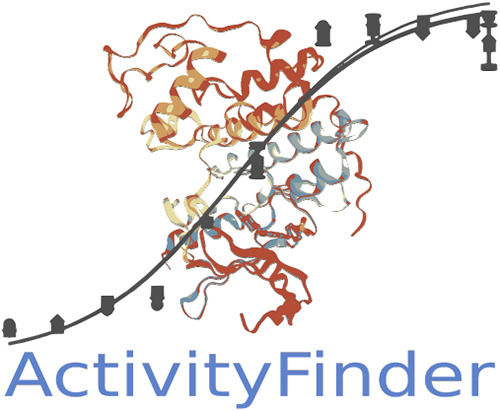

## Introduction

The predictive power of computational models depends largely on the quantity and quality of available training data. Gathering sufficient high-quality data to train models for predicting biomedical properties remains a highly labor-intensive and manual task. For six decades, models have been developed to categorize the chemical structure of compounds as active or inactive, or to quantitatively assess their activity in pharmacological assays.^1–4^ Empirical energy calculations of protein–ligand complexes and their validation with experimental values have been performed for at least five decades.^5,6^ Since then, many groups have addressed the problem of predicting the conformation and location of a ligand when bound to a host system (pose prediction) by molecular docking as well as the potency of a ligand interacting with a protein (scoring). Early efforts of ligand-host modeling had to build their own data sets from scratch using current literature in order to be able to develop models that are able to predict the properties of a complex.^7–27^ Relevant PDB^28^ entries had to be identified and the corresponding bioactivity data extracted from the primary literature. However, as advancements in computing power facilitated the creation of more complex models in drug design, more researchers became involved in model development, and the necessity for dedicated, standardized, high-quality data set resources became increasingly clear. As a result, several parties published their internal data curation efforts or began to systematically curate the data available in the PDB. AffinDB,^29^ BindingDB,^30–32^ BindingMOAD,^33^ Ligand-Protein-Database (LPDB),^34^ PDBbind,^35–37^ and Protein–Ligand-Database (PLD)^38^ are the most prominent examples of those efforts that considered structural as well as bioactivity data. These databases became important resources for the modeling community and have enabled a plethora of scoring functions across the whole methodological spectrum^39^ of empirical,^40^ physics-based,^41–44^ descriptor-based,^45–61^ consensus,^62,63^ and hybrid scoring functions^44,64–66^ as well as knowledge-based potentials.^15,18,67^ In addition to supporting initial development, the scientific community and model developers have thoroughly evaluated many docking and scoring tools based on these data resources.^68–74^ Additionally, since the mid-2000s, developments in machine learning have shown that deep learning, in particular, requires a substantial amount of data, far more than what is required for classical model development.^75–77^ Thus, it is undeniable that such data collections have a significant positive impact on the development of scoring functions. But only three data sets have been regularly updated since their inception: BindingDB, BindingMOAD, and PDBbind. Worse still, the number of publicly accessible databases has declined even further in recent years. The team behind BindingMOAD decided to cease their efforts,^78^ and PDBbind has changed access to their data to a per-release subscription model.

Hence, the problem persists, collecting and curating high-quality data sets for computational chemical modeling is a labor-intensive task that does not scale well.^30–33,78^ It is, therefore, highly desirable to develop strategies that automate data curation as much as possible to reduce the manual work and improve scalability. This automation must not come at the expense of the quality of the curated data set. Both the size and quality of training data are equally important.^79–83^ As a result, it is imperative that when creating a more extensive data set based on heterogeneous resources, there has to be emphasis on the foundation upon which the connection is made. The quality of the biological target structure is a key factor. Equally important is ensuring that the biological entity studied in a structural experiment matches those examined in wet-lab activity experiments. Lastly, careful attention must be given to the exact form of the, for example, stereochemical configuration, preferred protomer, and tautomeric state, of the agent whose activity is measured.

Two currently underutilized resources could strengthen the data foundation for developing models requiring both structural and bioactivity assay data: First, pure bioactivity databases^30,84–92^ and second, proprietary data sets. Pure bioactivity databases cover a subset of protein–ligand complexes in the PDB with bioactivity data that is not identical to the manually curated data sets presented above.^93^ Due to the nature of proprietary data sets, it is impossible to measure the exact amount of data or how distinct they are from publicly available data sets. However, it is safe to say that each pharmaceutical company has its own subspace within the chemical space it explores using its in-house data, which differs from that of other pharmaceutical companies.^4,94^

To our knowledge, no method currently exists to connect databases of biological structures and bioactive small molecules from scratch in an integrated and automated, end-to-end manner while adhering to the aforementioned requirements. Currently, it is only possible to use the link between PDB complexes to targets and ligands of wet-lab experiments if they are in the public domain and have been processed by the ecosystem of PDB,^28,95^ EMBL-EBI,^96^ UniProt,^97,98^ NCBI,^99^ and other initiatives. The SIFTS process powers the integration of PDB entries with UniProt sequences.^100–103^ While others have described strategies to create a sequence mapping from PDB files to UniProt sequences,^104,105^ SIFTS is the only resource that has provided mappings with residue-level resolution continuously to this day. Unfortunately, none of the available descriptions of these strategies are detailed enough to reimplement them fully. All strategies share a common practice of using the BLAST service of UniProt to map sequences of PDB files to UniProt sequences based on a sequence identity cutoff. ‘Cutoffs vary between 70 and 95%. But while they all somehow utilize the information given in the ATOM and SEQRES records of a PDB file, and sometimes other records, to create a residue-level mapping to UniProt sequences, the exact details of their process are not shared.

Integrating small-molecule ligand data necessitates robust methods for structure normalization, compound identity matching, and identifier mapping. UniChem and ChEBI are authoritative sources for chemical structures, identifier mapping, and their biological roles in the public domain.^106–111^ InChI-strings and their keys^112,113^ are used as unique canonical mappings for chemical structures. While they are used for their unique canonical representation and standardized normalization, usage of standard InChIs might lead to incomplete association of structures between two resources. Notably, a differing protonation state, tautomeric form, and salt are possible reasons for a miss. Dedicated curation pipelines can mitigate some of those issues, like those used at ChEMBL.^114^ Other approaches are nonstandard InChI-strings, canonical USMILES, or graph algorithms. Recently, PDBe, ChEMBL, and CCDC published a new resource, BioChemGraph,^115^ which presents links between published entries of PDB, ChEMBL, and CCDC in a machine-readable format. Those links are based on the services and resources they have developed over the past decades, using InChI and UniProt-based links between chemical structures and biological targets. Other parties, external to the EBI ecosystem, use the same approach to connect PDB protein–ligand complexes with ChEMBL assays.^93,116–121^

We developed ActivityFinder, a fully integrated and automated solution for linking crystal structures of protein–ligand complexes to wet-lab experimental values based on sequence and chemical identity. The approach uses crystal structures in the form of PDB files and bioactivity data from an SQL database. No other resources are needed, ensuring the privacy and locality of data and computing. Furthermore, we enable scientists to accurately estimate the quality of the cross-link between bioactivity data and 3D models of protein–ligand complexes using % sequence identity, mutation tracking in the binding site, and multiple small molecule matching levels. As part of our validation strategy, we applied our approach to the protein–ligand complexes published in the PDB and bioactivities published in ChEMBL. This allows us to gauge the accuracy and precision of our newly developed method by both manual investigation and a comparison on a larger scale with the results published by BioChemGraph.^115^

This cross-link results in structure-bioactivity data for 20197 PDB structures, 13734 PDB ligands, 17829 ChEMBL ligands and 2585 ChEMBL targets and over 1 million unique data points.

## Methods

ActivityFinder has been developed to search bioactivity databases for experimental values where the ligand and protein match those of the one reported in a PDB file. Results equal or similar to the query are compiled, accurately specifying the differences between the complex structure and the property measured in the assay. Differences are determined based on the sequence of the macromolecule and the structure of the ligand. Rapid searches are facilitated by a PostgreSQL database that records the associations of the entities of the crystallographic experiment and the bioactivity database. For the sake of simplicity, the methodology is described using PDB files and a recent ChEMBL PostgreSQL database dump. However, the strategies and methods covered here can be used regardless of the bioactivity database or file format, provided they contain structural information about the ligand, sequence details of the target, and experimental values linked to a protein–ligand complex.

ActivityFinder performs two separate tasks to link crystallographically resolved structures with bioactivity experiments. First, an ActivityDB instance is created from PDB files and a PostgreSQL dump of the bioactivity database. During database creation, sequence data and ligand data are processed separately. The process is illustrated in Figure 1 and will be discussed in greater detail in the subsequent sections. Second, the database, an ActivityDB instance, is queried to retrieve bioactivity information for a ligand, sequence, or query complex that has been part of the database creation step. The following sections provide a more detailed explanation of the database creation and query process. For its description, we use the PDB keyword terminology as documented in web resources of the PDB.^122^

**Figure 1:**
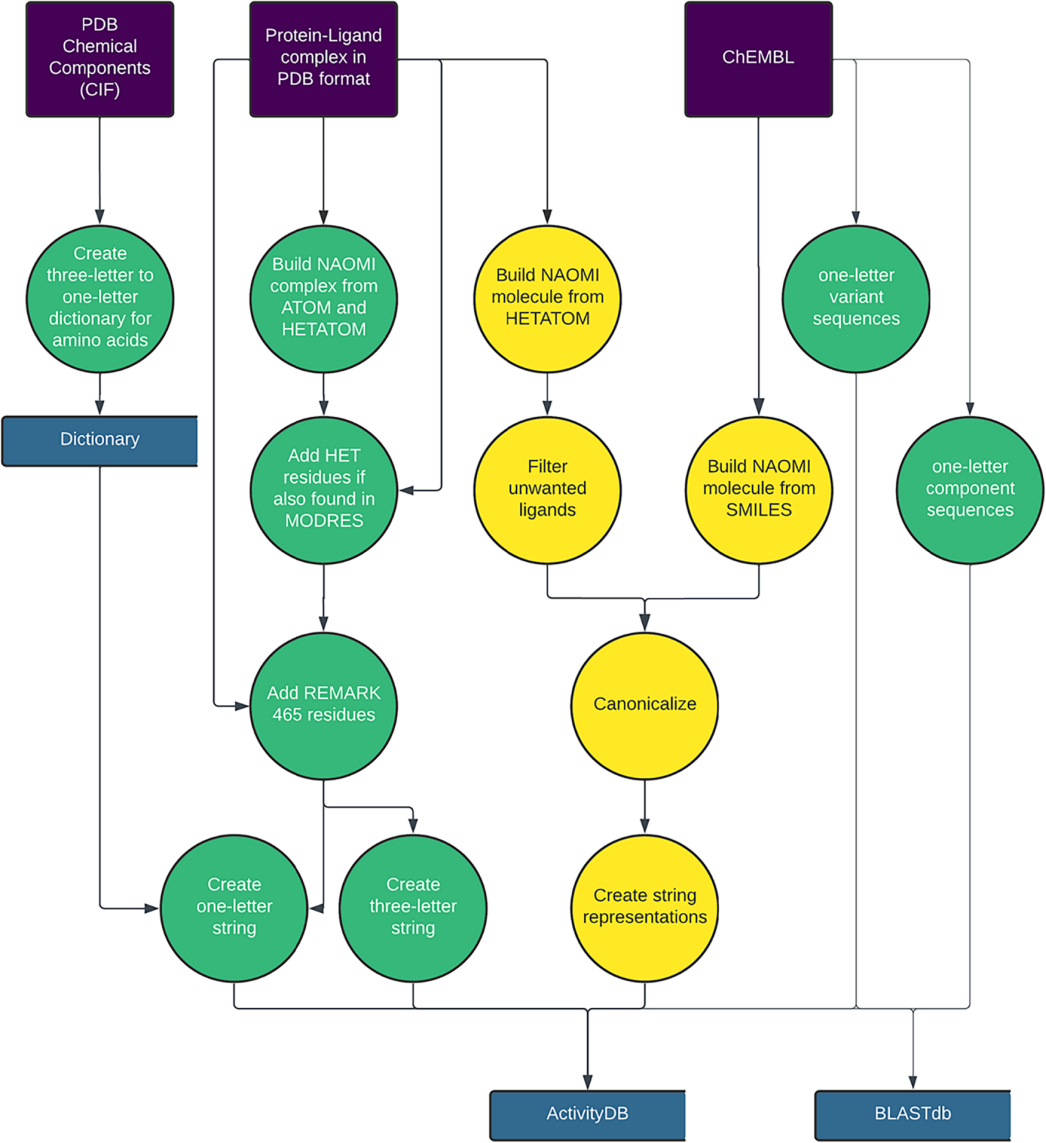
Data resources external to our process are colored purple, persistent storage formats are blue, ligand data are yellow, and target sequence data are green.

### Creating ActivityDB

The database is created in three steps. First, data that can be handled separately is extracted from a set of PDB files and a ChEMBL database dump and then recorded in a new database—an ActivityDB instance. Second, the biological and chemical entities that have been recorded are cross-referenced based on sequence alignments and chemical similarity of small molecules. Third, data from ChEMBL that cannot be copied verbatim but requires processing of PDB and ChEMBL data are identified and integrated into the ActivityDB instance. This pertains to everything that relies on sequence alignments and the similarity of chemical structures. The general data flow proceeds from sequence alignment to target identification, assay identification, and ultimately, the identification of activities and chemical structures. Following such a hierarchical process minimizes the data transferred from ChEMBL to the relevant subset defined by the complexes in the PDB input files.

The database creation workflow starts with parsing information presented as PDB files into NAOMI^123^ data structures for biological and chemical elements. The data structure of the macromolecule is derived from the PDB records ATOM, MODRES, HET, and REMARK465. A one-letter and a three-character string are generated for each chain from the internal data structure and stored in the database. In addition to the essential ATOM records, we also extract the sequences represented by the SEQRES and SEQADV records. The chains of the protein data structure and their variants are the basis for the sequence handling throughout the database creation and alignment workflow. The section below about sequences provides more information on how they are created.

Ligands are built from HETATM records as previously described^123^ and filtered for unwanted ligands (complete list in Table S3 in the Supporting Information). The remaining small molecules are standardized by retaining only the largest component (desalting), finding the canonical tautomer and the canonical protonation state, and scoring the localization of bonds.^123,124^ Isotopes are collapsed to the most prevalent form as they rarely affect the resulting bioactivity. Six string representations are created from the canonical NAOMI representation of the molecule and stored in the database: InChI, InChIKey, canonical SMILES with stereo information, canonical SMILES without stereo information, InChI connection and hydrogen layer, InChI connection layer. The string representations are covered in detail in the section on ligand processing below.

ChEMBL data is extracted from the tables of a PostgreSQL database dump downloaded from an FTP site.^125^ Data extracted includes small molecule data (COMPOUND_STRUCTURES,MOLECULE_DICTIONARY), sequence data (VARIANT_SEQUENCES, COMPONENT_SEQUENCES), target data (TARGET_DICTIONARY, TARGET_COMPONENTS), assay data (ASSAYS), and activity data (ACTIVITIES). Tables with target data are normalized into a single target table in the ActivityDB, which is connected to the main sequence table of the ActivityDB instance. The main sequence table is created by normalizing tables with sequence data into a single sequence table with two child tables containing meta-information about the sequences for each of the Component Sequence and Variant Sequence types.

A BLAST database is created from the sequences of each sequence type. Subsequently, these BLAST databases are queried with PDB sequences to identify targets shared between PDB and ChEMBL. We use optimal global pairwise alignments to track mutations and mismatches for each sequence of a PDB entry against relevant ChEMBL sequences, as detailed further below. Those ChEMBL bioactivity entries for which we could associate the target with a PDB entry are copied verbatim to the ActivityDB instance. Chemical structures, in the form of SMILES strings, are extracted, and NAOMI representations are generated based on them. These representations go through the same standardization process as PDB ligands. Finally, the five string representations are created for each remaining structure and stored in the ActivityDB instance, as is done for PDB ligands. The following sections cover the steps needed to create and query an ActivityDB in greater detail.

### ChEMBL and PDB Sequence Types

This section covers the types of sequences found in ChEMBL and PDB, as well as the details of sequence matching techniques applied.

Each amino acid can be represented by either a one-letter or three-character identifier. ChEMBL sequences use the one-letter notation. PDB sequences are composed of three-character amino acid identifiers. To map biological entities from PDB entries to ChEMBL target IDs, we need to understand the relationships among the different sequences contained in a PDB file and how they relate to sequences used in ChEMBL. This includes mapping the three-character amino acid identifiers in PDB files to the one-letter representations used in ChEMBL. Table 1 shows the exhaustive list of alphabets used to systematically describe the sequences considered in this article. For any alphabet Σ, we write Σ^+^ for the set of all finite, nonempty strings (sequences) over Σ.

**Table 1:** Alphabets for Amino-Acid Sequences Including Extensions by Gap-Symbols

alphabet	description
S	25 one-letter uppercase characters (i.e., A–Z excluding J)
$S_{x}$	S ∪ {x}; additionally includes x for unknown/mapped residues
$S_{g}$	$S_{x}\cup \left\{⊥\right\}$ ; additional gap symbol (rendered as “–”) in alignments
T	892 three-letter amino acid identifiers used in PDB
$T_{g}$	T∪{⊥} ; additional gap symbol (rendered as “—”) in alignments

ChEMBL stores two sequence types, Component Sequences and Variant Sequences, as strings $s\in S^{+}$ . Every target in ChEMBL comprises one or more Component Sequences. A single Component Sequence represents a single protein, while protein complexes or protein families comprise multiple Component Sequences. Component Sequences are sourced from UniProt and incorporated into the ChEMBL database after ChEMBL expert curators correctly identified the associations of ChEMBL target and UniProt sequence.^126^

In some cases, the authors of the primary literature on which ChEMBL relies on publish sequence information on the assay target along with the bioactivity data. In these cases, a Variant Sequence can be derived from a Component Sequence, annotating the authors’ changes to the UniProt sequence.^127–129^ Therefore, a Variant Sequence does not represent the general biological entity as a UniProt sequence does. Instead, it is specifically related to the experimental conditions of the bioactivity assay. Using Variant Sequences in our analysis improves our accuracy of mapping constructs of biological entities between experiments published in the PDB and ChEMBL assays.

The amino acid sequence of the structurally resolved macromolecule is found in the ATOM record of a PDB file. In cases where a crystallization experiment leads to an incomplete sequence representation of the biological entity being studied, the MODRES and REMARK465 records may be used to supplement missing ATOM residues if present. The SEQRES record represents the intended sequence of the biological construct used in the crystallographic experiment. In this context, *intended* refers to the sequence that the researchers designed and prepared for the study. It includes all expected residues: full-length polypeptides, modified residues, expression tags, and other components, even if not structurally observed.^130^ Finally, entries from the SEQADV record can be used to document differences between the SEQRES sequence and a sequence of a reference database like UniProt. ATOM, SEQRES, and SEQADV sequences are all members of $T^{+}$ . For the remainder of the document, we will refer to the sequence representations as ATOM, SEQRES, and SEQADV sequences. Table 2 summarizes the sequence types as used in this article.

**Table 2:** Sequence Types as Used in This Article

sequence type	set	description
ATOM	$T^{+}$	Sequence of amino acids actually observed in the crystallographic experiment.
SEQRES	$T^{+}$	A sequence that represents the complete, intended sequence.
SEQADV	$T^{+}$	Sequence created from the SEQRES sequence by applying the reported amino-acid changes of the SEQADV records to reconstruct the sequence of a reference database like UniProt.
Component Sequence	$S^{+}$	The sequence for a single molecular component (usually one protein subunit, isoform, or other macromolecular chain) that forms part of a curated biological target. A curated UniProt sequence.
Variant Sequence	$S^{+}$	ChEMBL reconstructed sequence used in the experiment by applying the reported amino-acid changes to a UniProt canonical sequence

PDB publishes the chemical component resource,^131^ that maps every three-character ID occurring in the PDB to a one-letter ID if possible. PDB chains yield sequences $t\in T^{+}$ from ATOM, SEQRES, or SEQADV records. We translate each $t\in T^{+}$ to a sequence $s\in S_{x}^{+}$ using a dictionary derived from the PDB chemical component dictionary; elements from T not present in the dictionary are translated to the symbol x. Users can supply three-letter IDs not included in this standard dictionary to avoid ambiguity.

The dictionary currently contains 892 mappings of three-letter IDs to their corresponding one-letter representations. Twenty mappings are attributed to the translation of canonical amino acids. 766 mappings cover translations from noncanonical amino acids to a single-letter code of a canonical amino acid, and 106 mappings cover the translation of noncanonical amino acids to a single-letter code of a noncanonical amino acid (X, U, O, B, Z). The histogram in Figure 2 shows for each element a∈S the number of three-letter amino acid identifiers mapped to it. The complete dictionary of three-character to one-letter identifiers is provided in the accompanying Supporting Information (amino_acid_mapping.csv). Since this translation results in a considerable loss of information, it is postponed for as long as possible when building an ActivityDB instance.

**Figure 2:**
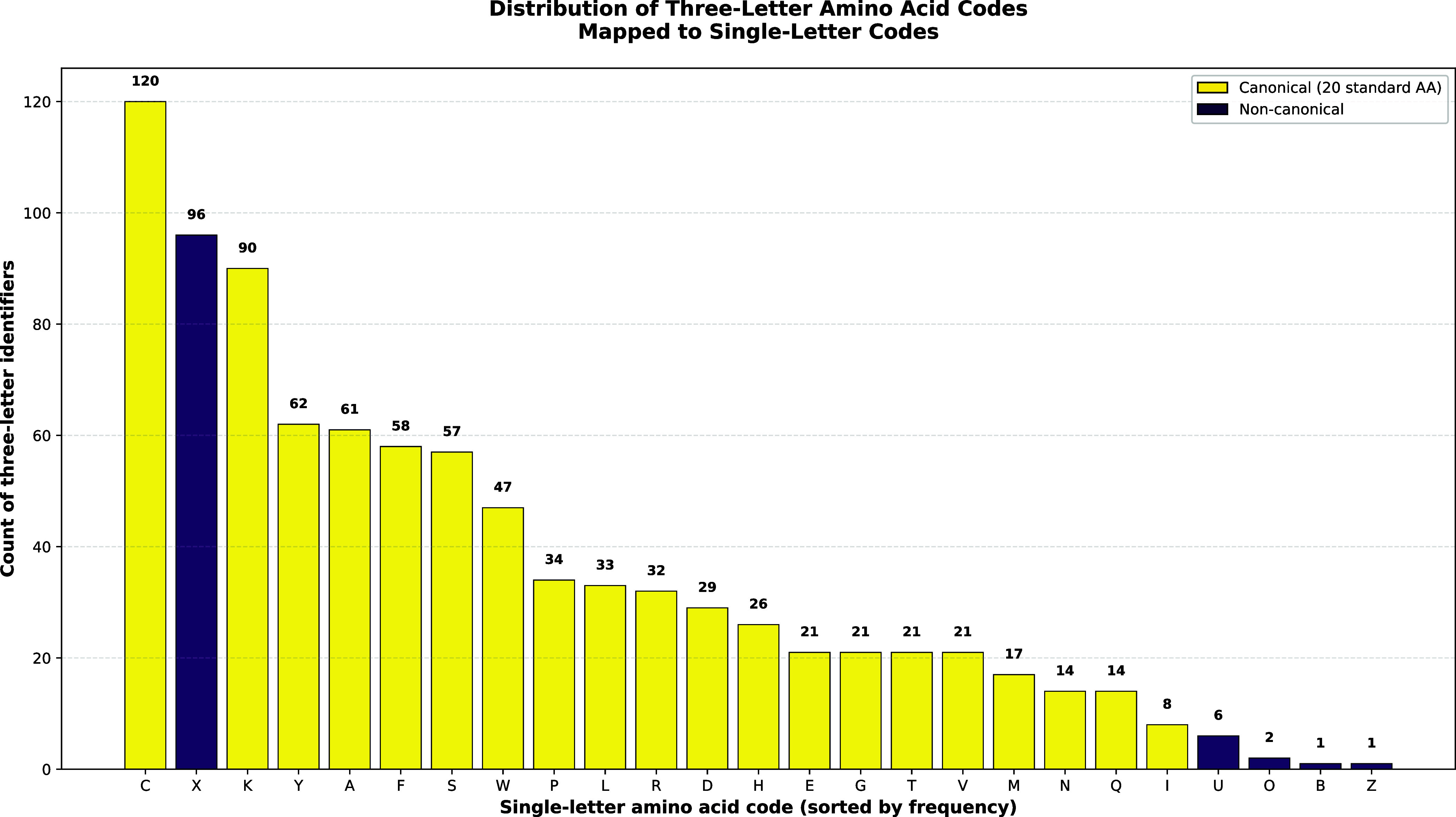
Frequency distribution of PDB residue identifiers mapped to IUPAC single-letter amino acid codes. Each bar represents the number of distinct three-letter residue identifiers mapped to a specific single-letter code, arranged in descending order of frequency. Yellow bars represent the 20 canonical amino acids, while purple bars indicate noncanonical or chemically modified residues. The data set consists of 892 three-letter identifiers (corresponding to 25 single-letter codes) extracted from the RCSB PDB Chemical Component Dictionary (accessed July 29, 2025).

### Target Mapping by Sequence Alignments

Matching biological entities between ChEMBL and PDB is achieved by running a BLAST search using a PDB sequence type as a query against a BLAST database created from a ChEMBL sequence type. Since a BLAST alignment can contain gaps,^132^ a subsequent optimal global alignment of the original PDB sequence and the query sequence identified in the BLAST result ensures accurate tracking of gaps that the BLAST search may have introduced. Furthermore, it reduces the impact of the information lost by translating the three-character amino acid sequences of the PDB to one-letter sequences of ChEMBL.

Several scenarios of interest require different pairings of PDB and ChEMBL sequence types. A scenario central to this work is to accurately map constructs of the same biological entity across different experiments. An unreleased PDB file typically contains limited information because it has not been processed by the PDB Europe SIFTS^101,102^ pipeline. Additional relevant information, as well as cross-references to other databases, are usually not annotated by the authors. As a result, we must be able to generate a link to bioactivity data solely based on the ATOM sequence and chemical structure created from information provided in the HETATM records. Considering SEQRES and SEQADV sequences beyond the ATOM sequence type allows us to answer two questions. First, what is the maximum sequence identity possible between the PDB macromolecule and the ChEMBL target? Second, how significant is the difference between relying solely on a mapping from PDB to ChEMBL based on identifiers and one based on sequence identity and chemical similarity?

As described earlier, every PDB file contains at least an ATOM sequence. We use the C++ implementation, BLAST+ ^133,134^ version 2.10.0.^132,135^ Searches are conducted using BLASTp with the default BLOSUM62 scoring matrix for the alignment process. Query sequences are formatted to FASTA^136^ from their NAOMI representation. Results are filtered by sequence identity. We record all BLAST results with a sequence identity range of 80 to 100% during the creation step of the database. Other parameters of the BLAST search are set to the default values of BLAST+. It is common^137–140^ to use a percent identity range of 90 to 100% when working with near identical sequences. A lower bound of 80% sequence identity ensures that sequence pairs can still be identified, if they only differ by engineered mutations, tags, or isoforms of the same gene.^101,105^ When submitting queries, users can set a sequence identity threshold larger than the threshold used upon database creation.

To correctly track modifications introduced to the query sequence during the BLAST search, an optimal global sequence alignment is performed between the ATOM sequence $t\in T^{+}$ and its one-letter representation $s\in S_{g}^{+}$ reported by the BLAST alignment. We employ the optimal global alignment strategy using an affine gap cost model.^141,142^ Cost values presented in Table 3 are optimized to accurately map crystallographic sequences to assay sequences of a target, which we assume represent the same biological entity. They have been determined based on a set of artificial sequences whose construction was guided by patterns observed in PDB data. The core assumption of this approach is that the sequences of the constructs are variations of a shared ancestor sequence. Tags at the start of an ATOM sequence are often split into single-residue matches if the relative cost of gap opening and gap extension is too low. Using the affine gap model with our chosen costs maximizes the gap length at the beginning of an alignment and minimizes cost. Additionally, it prevents trimming characters when there are spurious matches at the start of the sequences being compared. Furthermore, gaps caused by crystallographic artifacts or unresolved loops are identified by shifting residues to adjacent areas of matching residues, thereby creating a single large gap rather than multiple smaller ones within regions of mismatches.

**Table 3:** Cost of Edit Operations for the Affine Gap Model

symbol	cost type	value
*d*(*a, b*), *a* = *b*	Match	0
*d*(*a, b*), *a* ≠ *b*	Mismatch	45
*w*_open_	Gap opening	43
*w*_extend_	Gap extension	12
*w*_init_	Initial gap	5

To minimize bookkeeping during the workflow, the alignment is performed between the three-character representation of the ATOM sequence $t\in T^{+}$ and the one-letter amino acid query sequence $s\in S_{g}^{+}$ reported by the BLAST alignment using a lazy translation strategy. Translation is performed during the alignment process, which involves calculating the matrix using dynamic programming. Figure 3 visualizes the result of this process.

**Figure 3:**
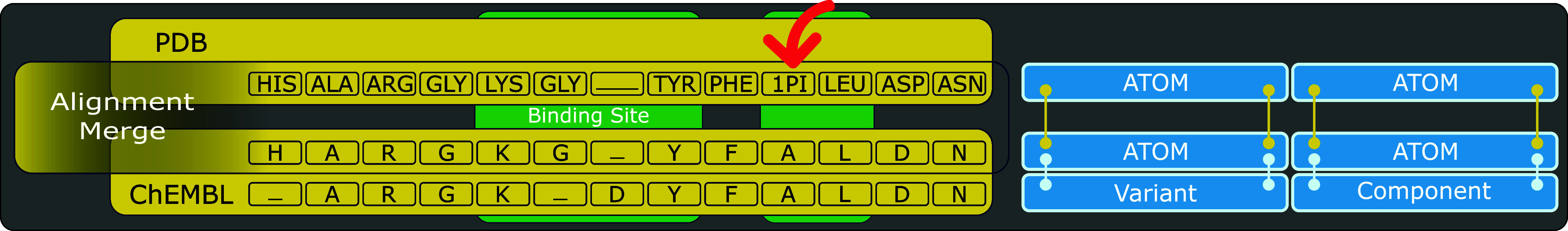
Image displays the situation after the BLAST search and after the global alignment for the ATOM sequence was performed. Sequences are drawn in yellow boxes with the source annotated at the box level. The Alignment Merge box shows a three-character sequence over $T_{g}$ and the corresponding sequence in one-letter code over alphabet $S_{g}$ . The latter sequence was part of a reference, which was identified by a BLASTp search of a ChEMBL database sequence, parts of which are shown on the line labeled ChEMBL. The alignment of the PDB sequence and the ChEMBL sequence (including the gaps) was obtained by our global alignment method. Blue boxes on the right annotate the sequence types used. Dumbbells show which pairs of sequences are used for the BLAST alignment (light blue) or optimal global alignment (yellow). The green boxes in the background indicate which amino acids are part of the binding site of the ligand. The red arrow highlights the noncanonical amino acid 1PI.

The case of SEQRES sequences is slightly more complicated than solely relying on ATOM sequences. SEQRES records are simple blocks of text that do not contain information other than the three-character identifiers. Consequently, the numbering scheme selected by the depositor and implemented in the ATOM record does not apply to residues of the SEQRES record. This is problematic in at least two ways. First, the amino acids that comprise the binding pocket are identified using the 3D model of the protein–ligand complex. This model is created from the information on the ATOM entries and, therefore, also follows their numbering. Hence, a mapping from ATOM records to SEQRES records is required to track structural features across sequence types. For example, suppose we identify a match between a SEQRES sequence and a ChEMBL sequence with a sequence identity of less than 100%. In that case, we can trace the nonidentical residues back to the three-dimensional structure despite the missing numbering scheme. Second, SEQADV records, which are discussed further below, annotate the residues that need to be replaced to obtain the reference sequence, using the same numbering scheme as the ATOM sequence. Since SEQRES residues are not numbered, we need to establish a mapping between ATOM and SEQRES residues to reconstruct the sequence of the reference database as given in the SEQADV records. The optimal alignment strategy described above can address both challenges by aligning the SEQRES and ATOM sequences.

**Figure 4:**
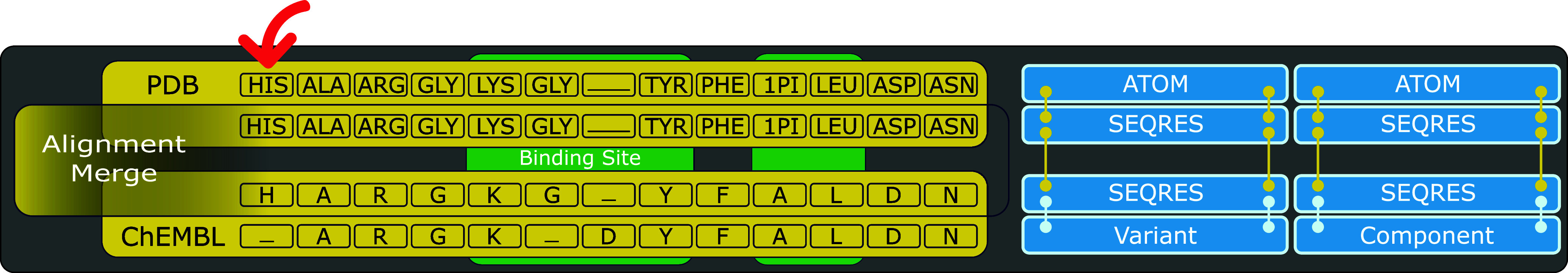
Image displays the situation after the BLAST search and alignment merge for the SEQRES sequence were performed. Sequences are drawn in the yellow boxes with the source annotated at the box level. The alignment in the PDB box results from an optimal global alignment with affine gap costs. The alignment in the ChEMBL box results from a BLAST search. The two sequences enclosed by the Alignment merge box have been used to produce the optimal alignment after the BLAST search. Blue boxes on the right annotate the sequence types used. Dumbbells show which pairs of sequences are used for the BLAST alignment (light blue) or optimal global alignment (yellow). The green boxes in the background indicate which amino acids are part of the binding site of the ligand. The red arrow highlights a HIS tag artifact of the crystallographic experiment.

In the following, processing a SEQRES sequence is identical to that of ATOM sequences as described above. This approach results in two alignments connected by the SEQRES sequence: a PDB internal alignment and a BLAST alignment. However, the two versions of the SEQRES sequence depend heavily on their counterparts in the respective alignments. Insertions, Deletions, and Replacements can differ significantly in the alignment with the ATOM sequence compared to the alignment produced by the BLAST search. To determine the importance of, e.g., a mutated residue in an ATOM sequence or a ChEMBL Variant Sequence, we need to track a residue across all four sequences (see Figure 4). We can again use an optimal global alignment to get the desired result, like when merging the ATOM sequence and its BLAST alignment above. The alignment is based on the SEQRES sequence $e\in T_{g}^{+}$ from the PDB internal alignment and the SEQRES sequence $b\in S_{g}^{+}$ from the BLAST alignment. Both *b* and *e* may already contain gaps. Thus, for the alignment merging process, we consider two occurrences of the symbol ⊥ (representing a gap) as matching symbols with cost 0. The third and last PDB sequence of interest is the SEQADV sequence. Most of the necessary methods have already been explained in previous paragraphs. Nonetheless, the SEQADV sequence is unique because it does not exist as a sequence in the PDB file itself. It has to be constructed from the information presented in the ATOM, SEQRES, and SEQADV records. SEQADV records are pairs of amino acid three-character identifiers. The first element of the pair lists the identifier of the SEQRES and, if present, the ATOM sequence that needs to be replaced with the identifier listed as the second element. Note that the index of the amino acid is based on the numbering scheme of the ATOM sequence, not that of the SEQRES sequence. Replacing amino acids in the ATOM sequence and not only in the SEQRES sequence is necessary, as any discrepancy between those two sequences would show up as nonidentical residues in the sequence identity calculation. Some residues annotated in the SEQADV records have not been resolved in the experiment and only need to be replaced in the SEQRES sequence. After this preparatory step, we can follow the same strategy as detailed for the SEQRES sequence above. Figure 5 visualizes the final product of the alignment merge.

**Figure 5:**
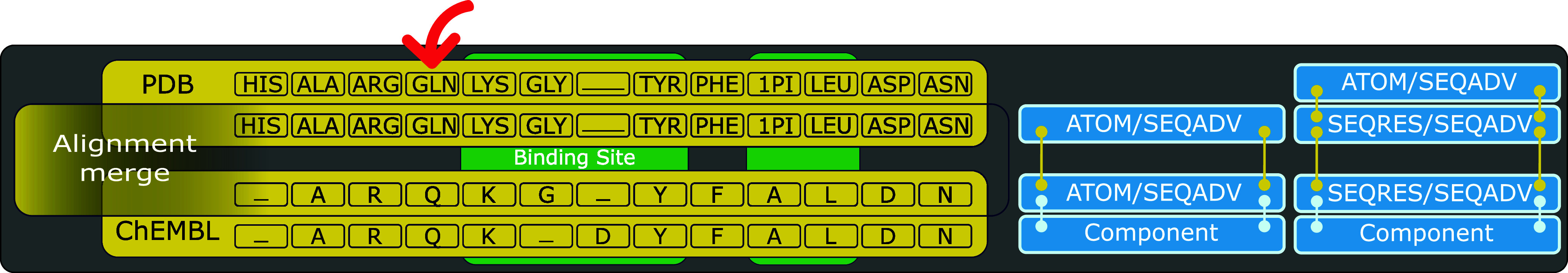
Image displays the situation after the BLAST search and alignment merge for the SEQADV sequence. Sequences are drawn in the yellow boxes with the source annotated at the box level. The alignment in the PDB box results from an optimal global alignment with affine gap cost. The alignment in the ChEMBL box results from a BLAST search. The two sequences enclosed by the Alignment merge box have been used to produce the optimal alignment after the BLAST search. Blue boxes on the right annotate the sequence types used. Dumbbells show which pairs of sequences are used for the BLAST alignment (light blue) or optimal global alignment (yellow). The green boxes in the background indicate which amino acids are part of the binding site of the ligand. The red arrow highlights the glutamine amino acid GLN (Q) that has replaced the glutamate GLU (E) as per SEQADV entry.

### Binding Site Mutations

While insertions, deletions, and mismatches can occur anywhere in the chains of a macromolecule relative to sequences of a ChEMBL target, we only explicitly register those relevant to the binding site. All residues and covalently bound molecules where at least one atom overlaps with or is fully enclosed within any sphere of diameter 6.5 Å around the center of a ligand atom are included in the binding site. Each valid ligand defines a binding site. All residues of all binding sites are recorded in the ActivityDB instance. During the query process, mutations, discovered during the alignments in the creation process and recorded in the database, are retrieved based on the PDB chain ID, residue name, sequence ID, and insertion code, and reported in the output file.

ActivityFinder has two query modes for target matching. The first, less specific one, considers every chain of the protein for a match with a ChEMBL target sequence. The second mode considers only those protein chains for a match that are part of the ligand’s binding site. This is another safeguard mechanism to ensure that the sequence of the ChEMBL target, and therefore the amino acid sequence relevant to the small molecule’s bioactivity, is the one present in the protein’s binding site as determined by the crystallographic experiment.

### Ligand

A NAOMI internal representation is created from the HETATM records of a PDB file alone, as described before.^123^ Protein chains that are less than six residues long are reclassified as ligands. In this version of ActivityFinder, we are not considering covalently bound ligands. The binding event may alter the ligand’s heavy-atom count, which renders the current approach invalid. A dedicated strategy must be developed to reliably map bound remnants of the initial ligand back to their original form. Currently, a PDB structure with no other ligand bound than a covalently attached one will be identified as an empty structure and not included when creating an ActivityDB instance. Like the ATOM record, the HETATM record can be expected to be present in all PDB files of protein–ligand complexes, regardless of whether they are part of the public body of official PDB structures, a proprietary data set, or have not yet been published and annotated with meta information by SIFTS. Records like HET, FORMUL, HETNAM, and HETSYN of the Heterogen section of PDB files are typically added by SIFTS. X-ray experiment artifacts like crystallization agents and buffers (PEG,^143^ salts,^144^ buffers^145^), cryoprotectants,^146^ heavy atoms and derivatives,^147^ and detergents and surfactants^148^ are removed. These artifacts are identified by their three-character ID issued by the PDB. We generally refer to this set of chemical structures as *unwanted ligands* in the context of our goal. Table S3 presents an exhaustive list of identifiers currently in use. The remaining structures are standardized by retaining only the largest component (desalting), identifying the canonical tautomer and canonical protonation state, removing existing isotopes, and scoring the localization of bonds and aromatic systems.^123,124^ Six string representations are created from the canonical NAOMI representation of the molecule and stored in the database. Compared to chemical structure matching based on maximum common substructure, or fingerprints, string representations offer two benefits for our application. First, they can be stored in a database and compared with little effort. Second, we are primarily interested in structural identity, rather than partially identical subsets of a structure. The purpose of ActivityFinder is to identify the appropriate bioactivity data for a protein–ligand complex given in a PDB file. Appropriate means, in terms of the ligand and our work, that the match is produced based on the identity of the set of heavy atoms, the connectivity of the heavy atoms, and the resulting stereochemistry. Differences of the reported structures in ionic states, salts, and associated charges are negligible in our case, as they are often a product of the nature of the experiment and have little predictive power in terms of whether the system described by a PDB file is the same as the one measured in an assay. Differences in tautomeric and protomeric states, on the other hand, are taken into account when matching two ligands. The goal of the canonization process is to generate a form of a chemical structure that, regardless of the input form, represents the unique form of that structure. NAOMI uses an atomistic valence state combination model to determine a canonical form of a chemical structure as described by Urbaczek et al.^123,124^ While the valence state constitutes a structured framework to handle varying forms of chemical structures composed of the organic subset of the periodic system, only monatomic forms of metals can be handled. Molecules containing covalently bound metals are not supported.

We have found that using six different forms of string representations is the best way to achieve the goals discussed. The six string representations can be divided into three broad categories. Identical molecules (InChI, InChIKey, canonical SMILES with stereo information), molecules only differing in stereochemistry, including racemic mixtures (canonical SMILES without stereo information), and molecules potentially identical that are outside the grasp of canonical SMILES with stereo information (InChI connection and hydrogen layer, InChI connection layer). In this version of ActivityFinder, we are not supporting enhanced stereochemistry in the form of CXSMILES.^149^ However, the representation constitutes an improvement over traditional SMILES that, among other features, reduces ambiguity when annotating mixtures or relative configurations of multiple stereocenters. We are planning to include enhanced stereochemistry in a future release of ActivityFinder. The first category allows us to identify identical chemical structures, as defined by NAOMI. Sometimes, stereochemical information is not annotated or available in both resources, PDB and ChEMBL. Importantly, many data points stored in ChEMBL are a result of measurements of racemic mixtures. Stereochemistry is not annotated in those cases, and we would otherwise miss them. Using category two enables us to cover those cases and not miss structures that only differ by their representation. Identical molecules with different InChI clippings and USMILES representations, due to limitations of cheminformatics algorithms, belong to category three. However, due to the nature of the clipped InChI representations, many molecules that are different also fall into this category. This enables scientists to find identical molecules for their specific PDB target that would otherwise be missed only due to limitations of the cheminformatics toolkit used. In reality, we only use five representations to record mappings between PDB and ChEMBL. InChI and InChIKey yield the same results, as we have not observed any collisions when generating the InChIKey from the InChI string. Therefore, it is safe to disregard the InChI string in the ligand mapping scenario.

## Results

Using ChEMBL 35 and a mirror of the PDB downloaded at 04.25.2025 ActivityFinder processed 226302 PDB structures in total. Of those, 88595 (39.15%) structures have been included in the resulting ActivityDB. Table 4 lists the reasons for the excluded or missing 137707 PDB structures. For 12 PDB structures, it was not possible to create valid sequence representations, reducing 88595 to 88583 (See Table S1). 88583 PDB structures account for 226082 chains (99961 sequences) of type ATOM and 225965 chains (43867 sequences) of type SEQRES. Note that several ATOM sequences might exist for a single SEQRES sequence, since only amino acids structurally resolved are contained. In total, only 59508 PDB IDs comprise a sequence of type SEQADV, resulting in 127421 chains (21183 sequences). Target sequences are selected from 10808 Component Sequences or 2645 Sequences and Variant Sequences. The median number of mutations introduced is one, with a maximum value of 20. Figure 6 shows the complete distribution. Finally, Table 5 summarizes the total sequence length by sequence type, which serves as a proxy for the complexity of the alignments.

**Figure 6:**
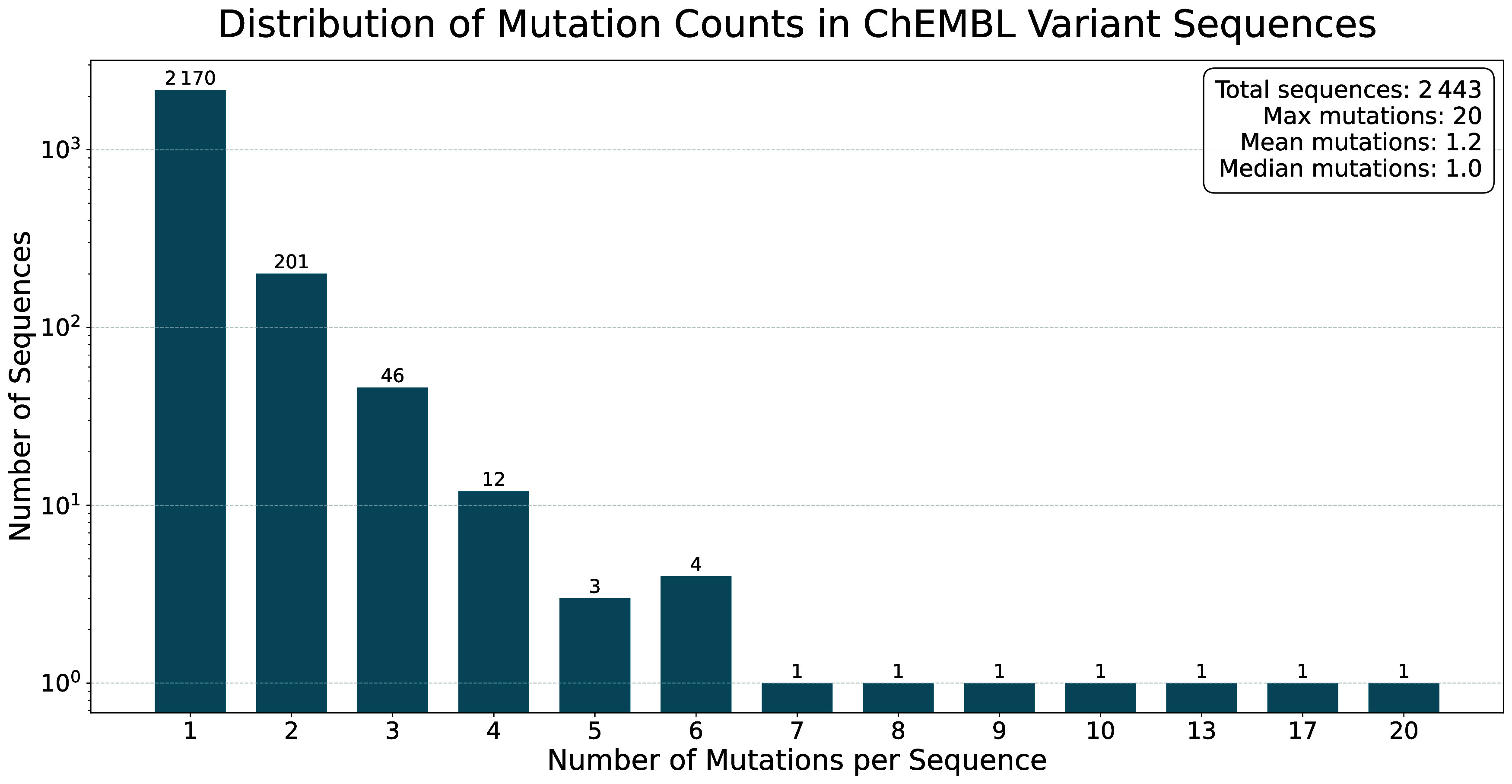
Distribution of mutations introduced into Variant Sequences. The majority of Variant Sequences (i.e., ≈89%) only contain one mutation relative to their parent Component Sequence.

**Table 4:** Number of Excluded or Missing PDB Files with Reasons Why They Are Not Included in the Final ActivityDB

no. of PDB files	percentage	reason why they are not included/filtered type of data^a^
99 616	72.34	no valid ligands for PDB structure. Either an empty component list, a metal, or an unwanted ligand.
22 262	16.17	Electron Microscopy
14 379	10.44	NMR Solution
843	0.61	Chain sequence shorter than 7 residues
245	0.18	Electron Crystallography
172	0.12	NMR Solid State
90	0.07	Neutron Diffraction
39	0.03	Fiber Diffraction
32	0.02	Solution Scattering
26	0.02	has no experimental data annotation
3		erroneous primary data

a Erroneous data refers to, for example, coordinates that would require atoms to have a certain valence and bond type combination that is not present.

**Table 5:** Total Sequence Length by Sequence Type^a^

sequence type	length
ATOM	64 120 795
SEQRES	67 955 879
SEQADV	41 366 834
Component Sequence	6 336 575
Variant Sequence	1 640 974

a Total sequence length is the length of all sequences of one type concatenated.

In total, we recorded 43231 ligands. This number is reduced to 34273 after deduplicating based on HET codes. Duplicates arise from the varying quality of ligand models in PDB structures. As expected, larger molecules like Chlorophyll A (CLA, 377), β-Carotene (BCR, 299), or Cardiolipin (CDL, 219), have many variants, while drugs like Ibrutinib (1E8, 3), Digoxin (DGX, 2), or Diazepam (DZP, 1), on the other hand, have rarely more than one variant. Figure 7 shows the distribution of structural variants for the unique count of 34273 HET codes. The complete list of values can be found in Table S4. 13702 HET codes map to 18655 unique chemical structures in ChEMBL. 9489590 bioactivity values are distributed across 757211 assays. Not every activity value is directly associated with a PDB ligand. Suppose an assay contains activity for a ligand that can be traced back to a PDB file. In that case, we pull the whole assay, its activity values, and chemical structures into the ActivityDB instance during the creation step.

**Figure 7:**
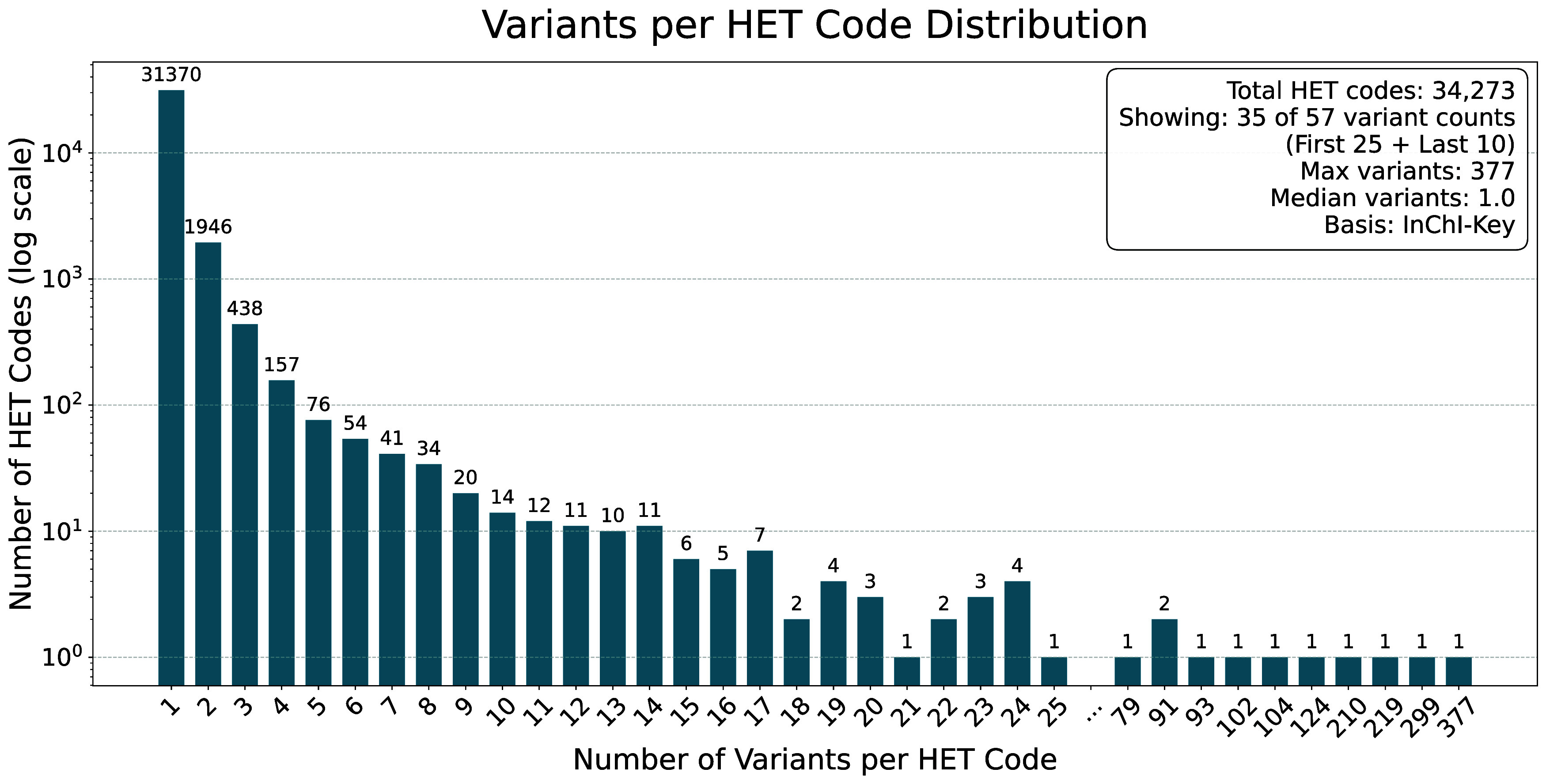
Distribution of variant structures per HET code found in the ActivityDB. Deduplication is based on InChIKey and is performed before any normalization of the molecules is performed. The values along the *x*-axis are not continuous. Values between 25 and 79 are omitted for the sake of brevity. The complete list of values can be found in Table S4. CLA has recorded the most variants at 377.

For each pair of sequences, we only keep the single highest-identity alignment. Then, we analyze unique (PDB sequence, ChEMBL sequence) best-hit pairs per sequence identity intervals. Identity intervals are disjoint: [100], [95, 100), [90, 95), [85, 90), and [80, 85). Figure 8 shows the associations of PDB sequence type and ChEMBL target sequence type and includes two ways of displaying the data: raw counts and normalized per-sequence-type counts, which are calculated by dividing by the total number of PDB-ChEMBL sequence pairs of each type combination (See Table S7 for an example). All percentages mentioned here are based on this compositional normalization, unless specified otherwise. For PDB-Component Sequence pairs, the interval counts (ATOM, SEQRES, SEQADV) of unique pairs in the 100% identity bin are 9786 (8.88%), 7354 (17.38%), 5857 (27.92%), with totals across all five bins of 110221, 42307, 20980, respectively. The [95,100) bin is the most populated bin for all three PDB sequence types. The combined high-identity share of the [95,100) and [100] bins is 42.33% for ATOM, 55.48% for SEQRES, and 58.12% for SEQADV. SEQADV concentrates a larger proportion specifically in the exact 100% bin. For PDB-Variant Sequence pairs, the interval counts in the 100% identity bin are 3185 (2.62%), 1769 (3.47%), 386 (8.99%), with totals across all five bins of 121488, 51051, 4295, respectively. Meanwhile, the [95,100) bin leads with 39.53, 54.57, and 70.59% for the three PDB sequence types.

**Figure 8:**
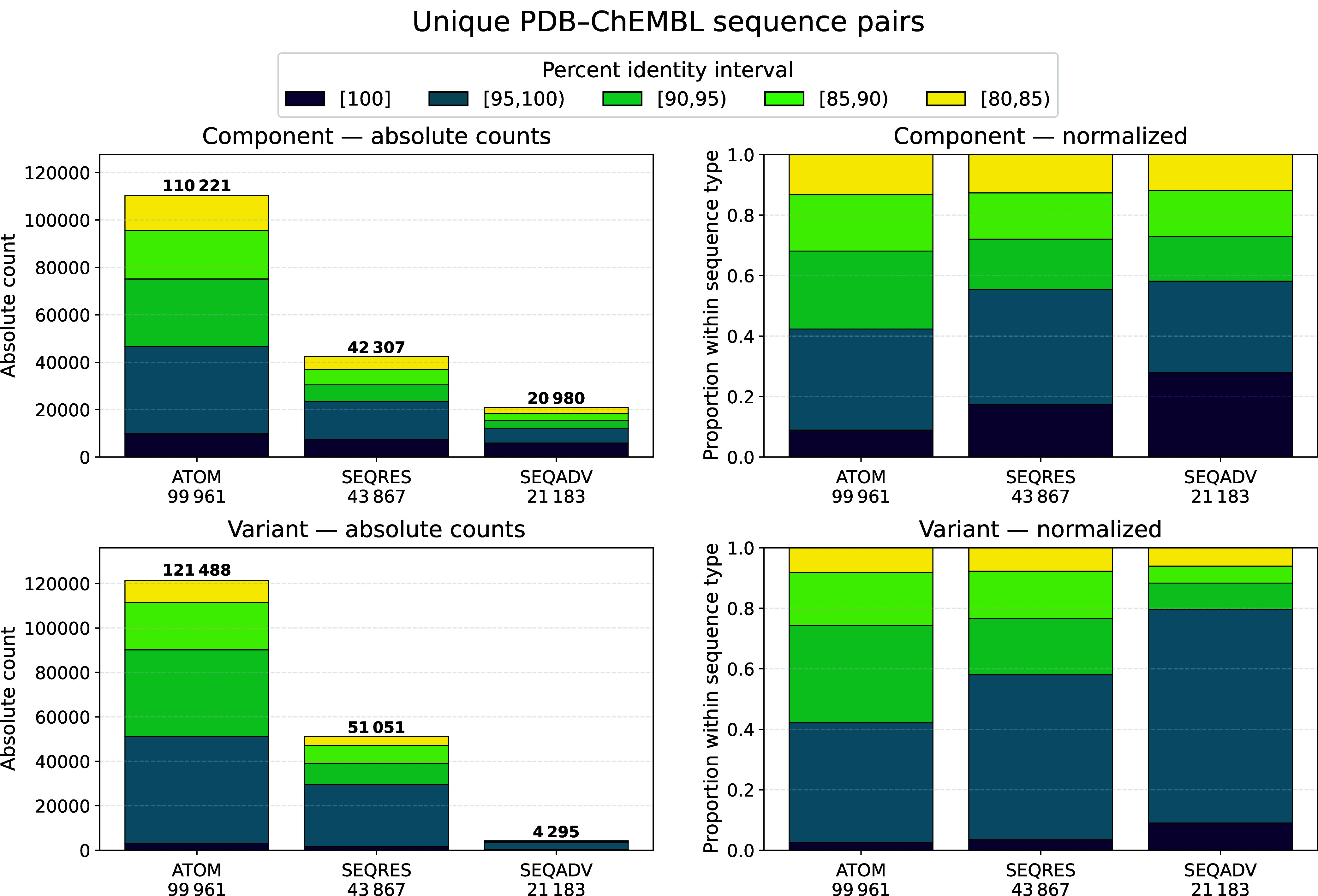
Results of the sequence type association analysis between PDB and ChEMBL sequences. The left column shows absolute counts. The right column shows the normalized values. Values for Component Sequence are displayed in the first row and values for Variant Sequence are displayed in the second row. The stacked bars in each subplot cover PDB sequences ATOM, SEQRES, and SEQADV from left to right. Totals of stacked values are annotated at the top of the bar, and unique sequence counts for each type are annotated at the bottom (x-ticks). Normalization is performed via the unique PDB sequence type count. For each PDB - ChEMBL sequence pair, only the pair with the highest sequence identity is recorded. If the same pair exists in a bucket of a lower sequence identity, it is disregarded. Exact values of the plot are attached in SI Tables S6 and S7.

In contrast to the analysis of sequence matching, we need to conduct a 2-fold analysis for chemical structures. First, an investigation needs to be performed that focuses solely on chemical similarity. Second, we are interested in how many pairs of chemical structures between PDB and ChEMBL remain after filtering for targets with measured activity values. Table 6 covers the results of the first analysis, and Figure 9 shows those of the second. The five string representations discussed earlier in the article are mapped to five confidence levels; InChIKey is level 5 and InChI connection layer is level 1. Across 72914 unique best PDB–ChEMBL small-molecule pairs (24913 distinct PDB ligands and 33644 distinct ChEMBL ligands drawn from universes of 43231 and 1688634, respectively), the confidence stratification is strongly bipartite: the highest confidence level (6) comprises only 25.6% of pairs but accounts for 47.3% of mapped PDB and 42.0% of mapped ChEMBL ligands. In contrast, the lowest confidence level (1) represents 49.9% of pairs but only 25.3 and 26.6% of the respective ligand diversities, indicating significant redundancy at low confidence. Distinct exclusivity patterns distinguish ligand sources further: 48% of mapped PDB ligands versus 72% of mapped ChEMBL ligands occur in exactly one confidence level, making multilevel reuse significantly higher on the PDB side. Intermediate levels (3, 2) offer moderate incremental diversity, while level 4 is a limited, highly selective group (1.4% of pairs) enriched with ligands unique to that level. Together, these distributions demonstrate that high-confidence mappings are diverse and ideal for strict structure-based analyses. Conversely, lower confidence levels lead to more pair counts but result in fewer gains in new ligand coverage. However, they can still be useful for exploratory or recall-focused purposes. Out of 21380 activity-supported ligand pairs with disjoint maximal-identity assignments, about 80% fall into the sequence identity bucket of 100%. Furthermore, there is sharp attrition across lower identity brackets. Approximately 11% for the range [95, 100), 4% for [90, 95), 2% for [85, 90), and 2% for [80, 85). Structural match confidence level aligns with this gradient. The highest confidence level accounts for 52% of pairs at 100% identity, decreasing to 37% in the 80 to 85% range. Meanwhile, the lowest confidence level increases from 17 to 36%, reducing the high-to-low confidence ratio from approximately 3 to almost equal. Middle confidence groups, especially level 3, remain stable at around 21 to 25% across intervals, indicating a strong intermediate tier. The concentration of pairs at exactly 100% identity (versus 95 to 99%) indicates that when activity-supported sequence alignments exist, they typically achieve perfect identity rather than near-perfect matches. Overall, the data show that most biologically supported mappings are sequence-redundant and have high structural confidence, with only a small tail where both identity and structural certainty decrease simultaneously.

**Figure 9:**
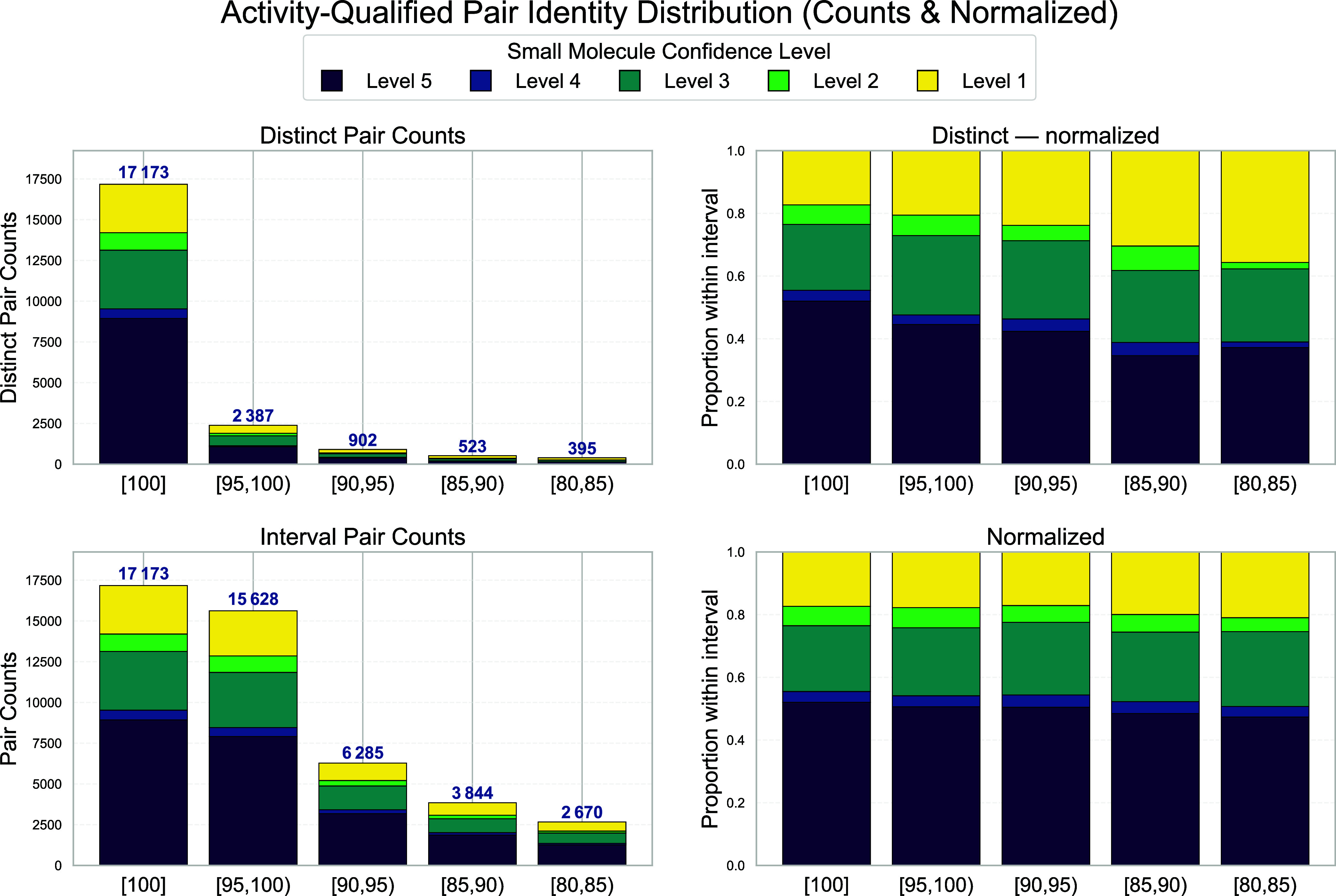
Distribution of activity-qualified PDB–ChEMBL ligand pairs across maximum BLAST sequence identity intervals, grouped by small molecule structural matching confidence levels. Each pair (pdblid, clid) is included only if supported by at least one ChEMBL activity record. In the top row, each pair appears exactly once, assigned to the interval containing its maximum observed sequence identity. In the bottom row, pairs appear in every interval where they have qualifying alignments, allowing multiple interval memberships per pair. On the left, absolute counts with interval totals labeled. On the right, normalized composition within each interval. Small molecule confidence levels indicate the quality of structural/identifier concordance: Level 5 (the highest, dark purple) is the InChIKey, while Level 1 is the connection layer of an InChI. The concentration at 100% identity (approximately 80% of distinct pairs) reflects high sequence conservation among activity-supported target mappings, with structural confidence declining systematically as sequence identity decreases.

**Table 6:** PDB-ChEMBL Chemical Structure Mapping Summary by Confidence Level (smmclid)^a^

	Map		PDB		ChEMBL		PDB_ex_		ChEMBL_ex_	
level	*n*	%	*n*	%	*n*	%	*n*	%univ	*n*	%univ
5	18 665	25.60	16514	47.32	16 589	41.95	9659	22.34	13 317	0.79
4	1035	1.42	780	2.23	922	2.33	536	1.24	792	0.05
3	12 880	17.66	6476	18.56	8658	21.90	2313	5.35	6067	0.36
2	3970	5.44	2292	6.57	2871	7.26	203	0.47	1854	0.11
1	36 364	49.87	8839	25.33	10 503	26.56	4040	9.35	6437	0.38

a Rows list unique strongest (pdblid, clid) structural pairs after canonical normalization; a pair matching multiple confidence criteria is retained only at its highest tier, so map counts are disjoint across levels. Column groups: map, PDB, ChEMBL, PDB_ex_, ChEMBL_ex_ (each, except exclusives, split into count *n* and percentage %). Map *n* = distinct (pdblid, clid) pairs at that level; map % = fraction of total pairs (disjoint sum). P/C *n* = distinct PDB/ChEMBL ligand IDs participating in that level’s pairs; ligands may appear in multiple levels via different partners. *P*_ex_/*C*_ex_ *n* = ligands that occur in exactly one smmclid level (level-exclusive); their percentages are computed against the complete ligand universes (PDB = 43231, ChEMBL = 1688634). All other percentages are computed as (level count/sum of per-level counts within the same column), meaning *P*_pct_/*C*_pct_ use a denominator that multi-counts ligands appearing in multiple levels (intentional to express distribution of appearances, not unique-union shares). Global unique mapped ligand unions: *P*_all_ = 24913, *C*_all_ = 33644. Absent smmclid levels indicate zero qualifying mappings.

Finally, we can analyze many unique PDB structures are covered by ChEMBL entries. Across both mapping routes (Component and Variant sequences combined), we identify 51330 unique quadruplets of PDB ID, PDB ligand ID, ChEMBL ligand ID, and ChEMBL target ID. Those are composed of 20197 unique PDB entries involving 13734 distinct PDB ligands with at least one bioactivity record in ChEMBL, spanning 2585 ChEMBL targets and 17829 distinct ChEMBL ligands. Restricting to the Component route alone yields 50486 quadruplets composed of 19874 PDB entries and 13491 PDB ligands linked to 2565 targets and 17528 ChEMBL ligands. The Variant route alone contributes 2385 quadruplets composed of 2273 PDB entries and 1028 PDB ligands associated with 195 targets and 1042 ChEMBL ligands. The intersection (pairs recoverable through both routes) comprises 2441 quadruplets with 1822 PDB entries and 715 PDB ligands mapping to 161 targets and 672 ChEMBL ligands, indicating that each route supplies substantial unique coverage with a modest shared core.

The underlying ActivityDB instance was built in one batch. PDB files were processed in parallel with 28 threads. Subsequent steps were performed sequentially due to their interdependence. All ChEMBL data selection, but for the component and Variant Sequences, is based on the filtered values from the PDB file processing. Within ChEMBL, data extraction follows the sequence of target, assay, activity, and chemical structure. Finally, to enable queries with an average run time of milliseconds, indices are created for crucial columns involved in the query logic. Table 7 coarsely lists the timings needed for each step.

**Table 7:** Timings of the Whole ActivityDB Creation Process^a^

		duration	
no.	step	minutes	hours
1	PDB file processing and data transfer to ActivityDB	71.10	1.19
2	Alignments and Alignment Merges	2157.80	35.96
3	Transferring assay data for set of target ids	5.71	0.10
4	Transferring activity data for set of assay ids	36.14	0.60
5	Transferring compound data for set of assay ids	1963.10	32.72
6	Creating database indices	3167.53	52.79
	Whole ActivityDB creation	7411.37	123.52

a PDB files were processed in 28 threads without hyperthreading. The host was powered by an Intel­(R) Xeon­(R) E5-4620 2.20 GHz CPU, provisioned with 128GB RAM and a 1TB SSD, running OpenSUSE Leap 15.3.

## Discussion

We aim to link a resolved protein–ligand complex to assay data that report both the ligand structure and the target sequence. We first focused on X-ray experiments in PDB format to establish a clean baseline. Relaxation to more types of experiments and formats will follow later. Three sequence abstractions matter: ATOM (only resolved coordinates), SEQRES (the deposited construct including unresolved or engineered parts), and SEQADV (the reconciled canonical form). Each brings a trade-off. ATOM sequences provide high structural confidence but lower sequence completeness, as they exclude unresolved regions, reducing overall alignment coverage compared to full target sequences. SEQRES sequences offer complete construct coverage but may include non-native elements (affinity tags, linkers, mutations) that reduce exact matches with wild-type target sequences. SEQADV sequences provide standardized, canonical representations by removing depositor-specific variations and documenting known discrepancies, offering the most consistent framework for cross-database comparisons.

Figure 8 summarizes the central sequence-focused insights captured in our analysis. Component (reference) mappings show a transparent gradient in perfect identity: ATOM < SEQRES < SEQADV. The observed proportions (approximately 9%, 17%, 28%) align with the construction logic: adding unresolved and engineered content (SEQRES) increases identity relative to the truncated coordinate-only ATOM, while reconciliation (SEQADV) removes extraneous differences and further enhances it. Variant mappings shift mass out of the 100% bin into the [95,100) interval, as single or few residue substitutions move pairs just below perfect while remaining near-identical. The much higher share of [95,100) for Variant–SEQADV pairs reflects curated point changes rather than broad divergence. A small fraction of Variant–SEQADV pairs still land at 100%. This could be due to genuine shared engineered mutations, alignments that exclude the mutated site, or rare curation drift that realigns historical differences.

Absolute counts differ from the analysis of proportions. ATOM yields the most significant raw number of sequence pairs because it includes many partial chains. These inflate lower identity intervals because missing or trimmed regions break global identity even when the overlapping core matches well. SEQADV covers a smaller universe but concentrates a higher fraction of perfect matches. Normalization removes absolute size effects from the interval profiles. As a result, the underlying contrast in composition becomes apparent.

A practical mapping strategy depends on the downstream goal. If the priority is a high-confidence canonical anchor for target linkage, start with SEQADV. To expand structurally resolved context while limiting the influx of lower-identity noise, a pragmatic layering could be to start with SEQADV for canonical anchoring, then add SEQRES to recover construct-level insertions. If instead the goal is to reconstruct exact experimental constructs, emphasize SEQRES–Component Sequence and SEQRES–Variant Sequence pairs because they retain tags and engineered regions that SEQADV normalizes away. Exclusive use of ATOM is not recommended when identity precision matters: it raises pair counts but dilutes perfect matches. Use SEQADV in cases where you need to pull in additional biological metadata. Exclusive SEQADV is precise but overlooks some almost perfectly usable contexts, and more importantly, obscures the differences between the actual construct used in the experiments, which renders the mapping invalid.

Figure 9 presents evidence in the same manner for chemical structures. We have 21380 activity-supported ligand pairs. Most of them (about 80%) are part of sequence pairs with exactly 100% sequence identity. The rest form a short tail: roughly 11% in [95,100), 4% in [90,95), 2% in [85,90), and 2% in [80,85). Adding all sub-100% intervals increases coverage by only about one-fifth, while the perfect identity set already dominates. Chemical structure match confidence follows the same pattern. At 100% sequence identity, structure pairs of the highest confidence level (InChIKey) account for a little over half of all pairs, while the lowest confidence (InChI connection layer) accounts for about one-sixth. In the lowest identity interval, those two bands are almost even. The middle confidence band stays steady across all intervals. As sequence identity drops, we mainly lose some top-confidence pairs and replace them with lower-confidence ones. That means we do not uncover a new class of mappings. The lower view considering pairs in more than the best sequence-identity bucket (lower row in Figure 9) confirms that loosening the identity threshold below 100% adds only a modest number of extra pairs. Extending below 95% adds little and shifts the mix toward lower structural certainty. An activity-first filter, combined with standardization, produces a large, clean core with perfect identity. Relaxing sequence identity to 95% is a reasonable upper bound for marginal gains. Going lower yields small numbers and more noise.

### Comparison to Identifier-Based Matching

By utilizing the links generated by BioChemGraph, we can compare the results produced by ActivityFinder to an identifier-based method. BioChemGraph constructs links between PDB and ChEMBL if the small molecules and protein targets are considered identical. Identity is established for protein targets by comparing UniProt identifiers and for small-molecules by comparing InChIKeys. As most matches between UniProt and PDB structures have a sequence identity exceeding the minimum of 80% set by ActivityFinder, fewer results should be expected from BioChemGraph. Furthermore, ActivityFinder allows matching of nonidentical molecules using a more inclusive approach. Additionally, our method enables the construction of multiple links for a single PDB ligand. For example, it can identify the R, S, and racemic forms of a ligand in ChEMBL at different matching quality levels if they occur in the data. Matching ligands that differ in stereochemistry or bond order may seem less useful than matching identical ligands. However, as mentioned in the ligand section, this data may be useful, especially if no identical ligands can be found otherwise. Similarly, while target matches with few or no mutations are most valuable, lower sequence identities can still offer useful insights, particularly when perfect matches are absent.

We employed the ActivityFinder filtering down to chains that are close to each ligand of interest to reduce false positives, resulting in slightly smaller numbers compared to the previous analysis. To compare BioChemGraph results with our results, we use triplets consisting of PDB ID, ChEMBL target ID, and ChEMBL molecule ID. Since ActivityFinder might find multiple links from a PDB ligand to ChEMBL ligands, a comparison of ligand HET codes would underestimate the amount of data linked with ActivityFinder. BioChemGraph reports 19333 unique triplets, ActivityFinder identifies 46287, and both approaches overlap on 14771. In Figure 10, a visual comparison of the unique and shared elements between both approaches is shown for PDBs, ChEMBL ligands, ChEMBL targets, and their combination.

**Figure 10:**
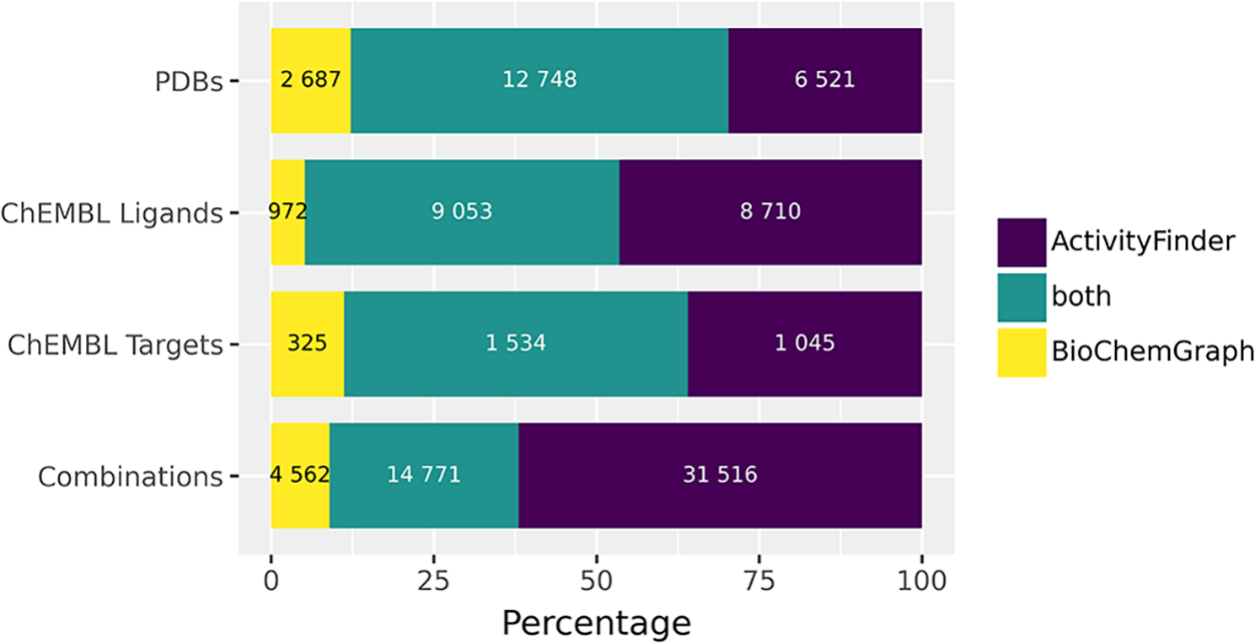
Visualization of the overlapping and exclusive unique results of BioChemGraph and ActivityFinder. Results are shown for PDB entries (top), ChEMBL ligands (upper middle), ChEMBL targets (lower middle), and a combination of all three (bottom). Purple area is instances only found by ActivityFinder, yellow only found by BioChemGraph and green by both approaches.

In the following paragraphs, we analyze in greater detail the reasons why triplets are found by only one approach.

#### Triplets Unique to BioChemGraph

The reasons why 4562 triplets are unique to BioChemGraph can be grouped into four categories (see Table 8). The highest number of missing links results from the fact that ActivityFinder currently only considers PDB structures based on X-ray experiments, while BioChemGraph also includes electron microscopy and NMR experiments. Differences in reproducing the ligand link proposed by BioChemGraph make up the second category. Building ligands directly from the HETATM records can result in a variety of different structures for the same HET code. Reasons can include incomplete modeling of ligands or variations in atom coordinates, leading to different derived chemical structures.^123^ BioChemGraph models those links based on HET codes, which represent the chemical used in the experiment. In contrast, ActivityFinder represents the compound model, i.e., the atomic structure visible and modeled by the crystallographer. Since both approaches might have their rationale in the eyes of users, the HET codes are stored in ActivityDB and are available for experienced users. However, they are currently not used for mapping activity values. Third, there are cases where our sequence-based approach cannot reproduce the link between a PDB protein and a ChEMBL target using UniProt IDs. For 50 of these links listed in BioChemGraph, the sequence identity is less than 80%.

**Table 8:** Number of Unique Triplets and Reasons Why ActivityFinder Does Not Reproduce a Link Proposed by BioChemGraph

reason	no. of unique triplets
PDB selection	3110
Ligand not reproduced	856
Target not reproduced	140
Workflow issues	456

The last category is due to workflow issues between BioChemGraph, ChEMBL, and the ActivityDB. A detailed explanation of the outlined categories is provided in Section 8 of the Supporting Information.

#### Overlap between BioChemGraph and ActivityFinder

The ActivityFinder and BioChemGraph produce many common links between PDB and ChEMBL. However, it becomes clear when analyzing the results produced by ActivityFinder that an identical UniProt identifier and HET code does not necessarily correspond to a sequence identity of 100% and an identical chemical structure. In 12100 triplets (81.92%), the ActivityFinder constructs the link with a sequence identity of at least 95% and identical chemical structure. BioChemGraph can link proteins to ChEMBL targets solely based on UniProt because of how UniProt IDs are annotated to PDB IDs during the SIFTS process. SIFTS cross-references biological entities even if the sequence identity is not 100% and annotates the differences in the PDB file. For example, a protein can be associated with a UniProt entry even after modification for the experiment has been performed, because it was the same biological entity that the UniProt ID represents before the modification. Furthermore, there are 1134 triplets (7.68%) in which the linked small molecules are not deemed identical and 1727 triplets (11.69%) where the sequence identity is below 95%. ActivityFinder does not link some chemical structures with identical InChIKeys, because the chemical structure constructed from the PDB coordinates differs from the structure encoded in the HET code and is only found due to the more lenient ligand matching levels. We do this because we believe that any differences between the modeled chemical structure and the HET code should be considered, as the modeled chemical structure is at the heart of the structural data used.

#### Triplets unique to ActivityFinder

The number of unique triplets of ChEMBL target, ligand, and PDB, depending on the molecule matching level and the best sequence identity, can be seen in Table 10. For 6575 cases, which is 34.01% of all triplets produced by BioChemGraph, ActivityFinder finds additional triplets of exact ligand matches with target matches of above 95% sequence identity. In 975 of the 6575 cases, the PDB, ChEMBL target, and ChEMBL molecule are all novel compared to the data from BioChemGraph, and only in 1082 of the 6575 cases are not novel. These novel triplets highlight that ActivityFinder is adding valuable new information for unseen PDB complexes, ChEMBL targets, ChEMBL molecules or all at the same time.

### Highlighting the Complexity of Structure-Bioactivity Linking

While linking bioactivity and structural data seems to be straightforward when the UniProt identifier and InChIKey match, the true complexity of the problem becomes clear only upon examining real-world examples. Therefore, we will discuss insightful examples to showcase the complexity of the situation and the advantages and disadvantages of our approach. An extensive analysis of all advantages, disadvantages, and exceptional cases we found comparing our method to identifier matching is available in the Supporting Information.

#### Low Sequence Identity Despite Matching UniProt Identifiers

UniProt identifiers are an excellent resource for finding exact matches of proteins in various databases or for easily referencing a specific protein sequence. However, in databases that allow or even encourage entries with sequence alterations like mutations, as is the case with PDB and ChEMBL, a simple matching by UniProt identifier understates the complexity of the problem. This becomes evident when we calculate the best possible sequence identity between all SEQRES sequences in a PDB structure to the UniProt sequence annotated by BioChemGraph (Figure 11). While most links constructed exhibit a very high sequence identity, there are 678 cases with percent identities below 90%, despite matching the same UniProt identifier. As we showcase in a following Section, even point mutations can have a drastic impact on bioactivity which often retain a higher sequence identity than 90%. In addition, there are rare cases with sequence identities below 40%. These cases are due to chimeric protein sequences containing multiple UniProt identifiers. One such case is the pair of PDB ID 5SYO^150^ and UniProt ID P32297 with a sequence identity of only 30.87%. While the assignment of multiple UniProt IDs is seldom, mutations to the sequence inside and beyond the binding pocket appear more frequently. In both cases, users need to be informed how well sequences in the bioactivity database and structural database match and where differences occur. This information enables users to understand if the link is of sufficient quality for their use case. This becomes even more important in the case of a mix of modifications, including Variant Sequences from ChEMBL, which are also derived from UniProt sequences.

**Figure 11:**
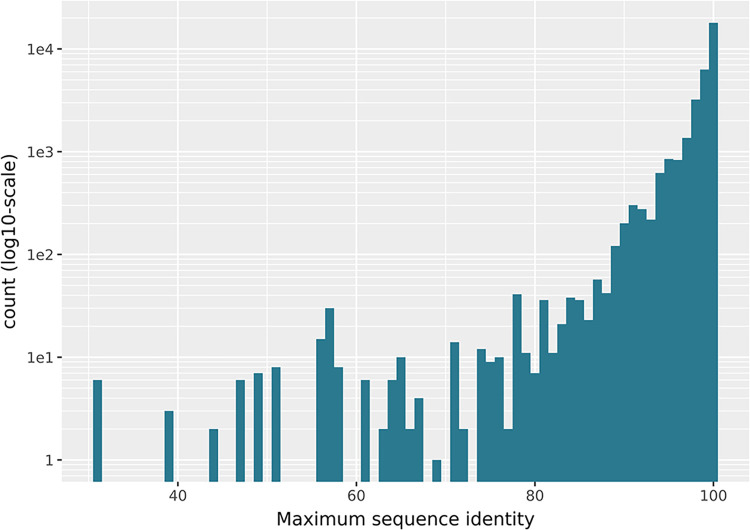
Maximum possible sequence identities calculated for all SEQRES sequences in the PDB structure, compared to the UniProt sequence annotated by BioChemGraph. Details of the sequence identity calculation can be found in the Supporting Information Section 8.4.

#### Differences in Modeled Small Molecules from Templates Associated with Annotated HET Codes

To accurately represent small molecules modeled in PDB structures, ActivityFinder derives structural information from atomic coordinates not relying on HET code annotations. Minor differences between the modeled small molecule and the annotated HET code may occur, for example, due to changes in bond order (e.g., HET code 5LO in 5EC9 ^151,152^ see Figure S6b) or incomplete ligand modeling, probably caused by missing electron density (e.g., HET code P8T in 6YQN^153,154^ or 2WE in 4PTC^155,156^ see Figure S2). Examples of drastic differences are HET code CQD in 4LMT^157^ where an originally aromatic system is not modeled planar (see Figure S6a), or the complex ligand MYC in 5HXC,^158,159^ which is just modeled as multiple different alkanes (see Figure S1). A special case is ligand BRH, where BioChemGraph matches the racemic ligand CHEMBL73698 (see Figure S6c). ActivityFinder also links to this ChEMBL molecule, but without chiral information. Although the string representation of ligand BRH is racemic, the modeled ligand is not, allowing ActivityFinder to estimate link quality accurately.

There are good arguments for modeling a chemical structure from coordinates reported in the PDB structure as well as relying on its HET code annotation. An argument for using the HET code is that small changes in the coordinates of modeled atoms can cause significant differences in the resulting chemical structure, such as a change in the derived bond order. Additionally, if you want to find where a chemical structure is bound in the PDB, searching with the HET code also yields structures in which the molecule is only partially built. On the other hand, discrepancies between the modeled and the HET code’s chemical structures can lead to inconsistencies and errors when analyzing or using them, particularly in an automated manner. Regardless of one’s opinion regarding the use of HET codes, it is crucial to make inconsistencies like these transparent so that scientists can decide whether the modeled structure is sufficient for their use case.

#### Complications of Molecule Matching via InChI or USMILES codes

There are further intricacies when matching molecules between databases with InChIs or USMILES. Discrepancies can occur in the string representations, even if they refer to molecules identical in the eyes of an experimentalist. ChEMBL already recognizes this through alternative molecule forms, for example, when two entries only differ by buffer agents or other additives. One example is the molecule pair CHEMBL1201774 and CHEMBL1201775, which differ by two hydrochlorides (see Figure S7a). While ActivityFinder links both molecules to H4B, BioChemGraph only links the first molecule to H4B, which is formally correct, but misses further available data. In the context of bioactivity integration, it is preferable to use the structure of the bioactive compound in isolation and avoid relying on a specific formulation of its substances.

Currently, nonstandard isotopes are not supported by the chemistry model used in NAOMI. When nonstandard isotopes are encountered during chemical structure processing, they are replaced with their predominant isotope. As a side effect, we link, for example, the HET code MC and CHEMBL3350473 (see Figure S7c). Since isotopes rarely affect bioactivity, ignoring isotopes results in further, reasonably associated data records. Lastly, while the normalization procedures implemented in different USMILES and, in particular, InChI work well on most molecules, there are some cases in which they fail, as noted in the technical documentation of InChI.^112^ An example of a molecule with differing InChI is the HET code MYC in 2O63,^160,161^ where a link is constructed to CHEMBL198468 (see Figure S5). This is a tautomeric form of CHEMBL164 that is not registered by BioChemGraph, due to a nonstandardized keto–enol tautomerism. We are able to link both tautomeric forms to this instance of MYC. However, in the current implementation of ActivityFinder, links like this are only found in the clipped InChI levels, which typically represent slightly different molecules. Typical examples of low-level ligand matches are provided in Figure S3. Therefore, finding similar molecules like this is only possible through manual investigation involving chemical knowledge.

#### Linking Equally Mutated Bioactivity and Structural Data

Taking all sequences in PDB and ChEMBL into consideration allows us to precisely track any mutations and differences between the respective sequences that a simpler approach would ignore. One example is the PDB 3QRJ,^162,163^ which is a structure of the Tyrosine-protein kinase ABL1 with the engineered mutation T315I in the binding site and is matched by BioChemGraph and ActivityFinder to the target CHEMBL1862 and the ligand 919 to CHEMBL1738757. The PDB’s ATOM and SEQRES sequence contain this mutation, but it is marked as an engineered mutation in the SEQADV records and, therefore, is not part of the linked UniProt identifier. When investigating the assays of this target, assay CHEMBL5108948 has a Variant Sequence with an identical mutation as is found in the PDB structure. Using this Variant Sequence, we get a sequence identity of 100% relative to SEQRES and 100%/98.10% relative to the ATOM sequence (Chain A/B). Additionally, as we specifically track any mutations in the active site, mutations toward the component sequence are annotated and provided to the user, along with the bioactivity data. Using the sequences above allows us to correctly link only the assay CHEMBL5108948 with 100% identity and no active site mutations. Mapping UniProt identifiers results in assigning five different assays, including CHEMBL5108948. However, the other four contain the critical active site mutation in the PDB that was not present when measuring the bioactivities. Interestingly, the bioactivity values measured without a Variant Sequence are both higher and smaller the one with Variant Sequence, even though the ones with higher numerical pChEMBL values are K_d_ measurements and not IC_50_. A similar case with a significant bioactivity gap is the Epidermal Growth Factor structure of ligand QFO (CHEMBL5173517) in 8D76,^164,165^ which is linked to the target CHEMBL203. Three point mutations are introduced in the structure, out of which two are corrected in assays CHEMBL5141217 and CHEMBL5141214. Compared to IC_50_ measurements without these point mutations (all over 1000 nM), the assays accounting for point mutations result in 4.8 and 0.1 nM.

#### Relevance of thorough Linking of Structure and Bioactivity Data

Relying solely on matching identifiers compared to our approach has two types of drawbacks. The first is that the linked data is not actually identical, despite being linked by identifiers. There are many cases in which the sequence identity is lower than most would expect given matching UniProt identifiers (see Figure 11), which is further showcased by the fact that 11.69% of triplets found by both approaches have a sequence identity of below 95% (see Table 9). Given the relevance of even point mutations to bioactivity data, changes like this are unacceptable when constructing high-quality data sets. InChI works almost perfectly for linking small molecules; we only found a single fringe case in which the normalization procedure did not suffice. But there are significant differences in the modeled and HET code defined molecules in PDB structures. In the overlap between the two methods, there are 1134/7.68% cases where our approach does not identify the molecules as identical. The second drawback of solely relying on matching identifiers is that only linking by identity could miss valuable data. Just using identical UniProt identifiers is too conservative, as we are ignoring those with very similar or identical sequences. When linking molecules with InChI, we neglect the linking of racemic mixtures or isotopic differences, although this linking might be beneficial. The number and quality of extra links created by ActivityFinder, as shown in Figure 10 or Table 10, attest to the amount of data that can be gained. Overall, 6575 extra links with above 95% sequence identity and identical molecules are missed when relying solely on identifier-based matching.

**Table 9:** Categories and Size of Quality Levels the Reproduced Links between BioChemGraph and ActivityFinder Fall Into^a^

molecule matching level	sequence identity (%)	no. of triplets
Identical	[100]	3383
	[95,100)	8717
	[80,95)	1537
Achiral	[100]	143
	[95,100)	301
	[80,95)	138
Potential	[100]	153
	[95,100)	347
	[80,95)	52

a Identical molecules fall into the InChIKey or canonical SMILES with stereo information molecule matching levels. Achiral falls into the canonical SMILES without stereo information level. Potential fall into the InChI connection and hydrogen layer and InChI connection layer levels.

**Table 10:** Triplets of PDB, ChEMBL Target, and Molecule Unique to ActivityFinder Depending on Their Ligand Matching and Sequence Identity^a^

molecule matching level	sequence identity (%)	no. of triplets
Identical	[100]	851
	[95,100)	5724
	[80,95)	6085
Achiral	[100]	1325
	[95,100)	5060
	[80,95)	2674
Potential	[100]	1934
	[95,100)	5177
	[80,95)	2686

a Identical molecules fall into the InChIKey or canonical SMILES with stereo information molecule matching levels. Achiral falls into the canonical SMILES without stereo information level. Potential fall into the InChI connection and hydrogen layer and InChI connection layer levels.

### Potential Improvements of ActivityFinder

ActivityFinder is the first tool that enables cross-linking of protein–ligand structure data to bioactivity data on the basis of the primary data types, the protein sequence and the small molecule chemical structure. While the prototype we present is already productive and creates very useful data resources, there is obviously room for further improvements.

To improve the method, one could further differentiate the canonical SMILES without stereo information ligand matching layer into cases where there is contradicting stereoinformation and cases in which the difference is due to an unspecified stereocenter. This would allow for separating cases of contradicting stereoisomers and matches to racemic ones.

Ligand processing from bioactivity databases could also be improved by annotating if a mixture was processed, as only the largest component of the mixture is then used. This would enable users to further restrict their resulting bioactivity data to assays that do not contain mixtures. Lastly, the process of reading ligands from their coordinates is only *almost* trivial, and with a data set as large as the PDB, there are undoubtedly cases in which the built chemical structure differs erroneously from the one associated with the HET code.

On the technical level, there are several options to improve the usability and sustainability of ActivityFinder. First of all, it would be helpful if the ActivityDB could be constructed with fewer restrictions on the used structures. Most importantly, this includes structures, for example, resolved by cryo-EM, but it further extends to apo structures. Finding ChEMBL targets for apo structures is currently possible with the target mode of ActivityFinder, but only if a complex exists in the ActivityDB that matches the corresponding ChEMBL targets. As ActivityFinder was designed for finding protein–ligand bioactivity data, apo structures were deemed less important, but the method could be generalized to allow for finding all apo pockets.

ActivityFinder is usually only run once on a combination of two databases, such that the computing times play a minor role. The current implementation takes about 123 h. This runtime can be significantly improved by adopting a more efficient table structure that is less normalized and requires fewer indices. Alignment calculation and alignment merges are currently in a first-pass implementation state as well. Furthermore, the implementation presently lacks an update functionality. As a consequence, ActivityDB has to be rebuilt in case new data becomes available in one or both databases.

## Conclusion

While the task of linking activity data and structural data may seem trivial at first glance, it presents numerous intricate challenges, which, if not accounted for, can introduce significant noise or even errors into derived data set or analysis. ActivityFinder addresses these challenges by employing rigorous sequence alignment, mutation tracking, and detailed ligand matching processes. It significantly improves upon traditional methods by automatically capturing and reporting subtle yet crucial variations such as binding-site mutations and discrepancies in ligand modeling. Reporting these intricacies is just as important as the link itself, so that scientists can decide whether the quality is sufficient for their specific needs. Furthermore, while we showcased the capabilities in the examples of ChEMBL and PDB, ActivityFinder enables linking any set of protein–ligand structures with wet-lab assays based on sequence and similarity of chemical structures. Therefore, it is not limited to these two databases or to databases within the public domain in general.

## Supplementary Material





## Data Availability

The ActivityFinder Prototype is available for licensing through the Naomi ChemBioSuite (at https://uhh.de/naomi). We are releasing the data set resulting from linking PDB and ChEMBL via the REST API of the ProteinsPlus^166^ platform and as a PostgreSQL database dump through the FDR of the University of Hamburg (at https://www.fdr.uni-hamburg.de/record/18143). The REST API has a SWAGGER documentation at https://proteins.plus/api/v2/ and a Jupyter Notebook accessing and all analysis code in this paper is given at https://github.com/rareylab/ActivityFinder_Analysis

## Notes

Additional methodological details and Supporting Information, including images of discussed molecules and more detailed discussion of the comparison to identifier-based matching (PDF)
Two CSV files detailing the amino acid mapping and the unwanted ligands (ZIP)
