# Supplementary material for: ActivityFinder: Toward the Fully Automatic Integration of Structural and Binding Affinity Data
Source: J Chem Inf Model. 2026 Jan 2;66(2):1013–34. doi: 10.1021/acs.jcim.5c02505 (PMC12848982; doi:10.1021/acs.jcim.5c02505)
Supplement: Supplementary file 1 [file ci5c02505_si_001.pdf]

Supporting Information: ActivityFinder: Towards the fully automatic integration of structural and binding affinity data

Emanuel S.R. Ehmki,[†,‡] Torben Gutermuth,[†,‡] Tobias Harren,[†] Stefan Kurtz,[†] and Matthias Rarey[*,†]

† *University of Hamburg, ZBH - Center for Bioinformatics, Albert Einstein Ring 8-10, 22761 Hamburg, Germany*

‡ *These authors contributed equally*

E-mail: matthias.rarey@uni-hamburg.de

1 Additional resources

We provide three additional resources for data and information. First, supporting data in the form of a machine-readable version of the unwanted ligands and amino acid mapping. Second, an FDR repository is available at `https://www.fdr.uni-hamburg.de/record/18143`, with all supporting data, including a database dump of the `ActivityDB`. Third, a GitHub repository at `https://github.com/rareylab/ActivityFinder_Analysis` includes all code used for the analysis in this paper, including a Jupyter notebook that details how to query the ProteinsPlus API for the ActivityFinder.

2 Additional details of PDB file processing

Table S1: PDB IDs without chain and sequence information in the ActivityDB.

Index	ID	Reason
1	1PGI	All residues UNK / ambiguous (CA trace placeholder)
2	2Z41	All residues UNK / ambiguous (CA trace placeholder)
3	2XSM	All residues UNK / ambiguous (CA trace placeholder)
4	1C51	All residues UNK / ambiguous (CA trace placeholder)
5	1HR3	All residues UNK / ambiguous (CA trace placeholder)
6	1PYK	All residues UNK / ambiguous (CA trace placeholder)
7	1XIA	All residues UNK / ambiguous (CA trace placeholder)
8	1KGA	All residues UNK / ambiguous (CA trace placeholder)
9	8RC7	Very short fragment (no robust sequence mapping)
10	1ILX	All residues UNK / ambiguous (CA trace placeholder)
11	1IVI	All residues UNK / ambiguous (CA trace placeholder)
12	8VPJ	Very short fragment (no robust sequence mapping)

3 Unwanted Ligands

Table S2: Common non-biological molecules found in crystallographic structures, grouped by functional category.

#	Category	Example Molecules	Function
1	Cryoprotectants	Glycerol (GOL), Ethylene Glycol (EDO), Dimethyl Sulfoxide (DMSO)	Prevent ice formation during cryocooling
2	Precipitating Agents	Polyethylene Glycol (PEG), Ammonium Sulfate (SO4)	Promote protein crystallization
3	Buffer Molecules	Tris (TRS), HEPES (EPE), MES (MES), MOPS (MOP)	Stabilize pH; may bind protein
4	Detergents	n-Dodecyl-β-D-maltoside (DDM), n-Octyl-β-D-glucopyranoside (BOG)	Solubilize membrane proteins
5	Reducing Agents	Dithiothreitol (DTT), β-Mercaptoethanol (BME)	Maintain cysteine residues in reduced form
6	Heavy Metal Ions and Compounds	Hg^{2+}, UO_2^{2+}, Pt^{2+}, Pb^{2+}, Au^{3+}	Used in phasing methods; bind proteins
7	Organic Solvents and Additives	2-Methyl-2,4-pentanediol (MPD), Isopropanol (IPA), Ethanol (ETOH)	Assist in crystallization or cryoprotection
8	Purification Tags and Linkers	Imidazole (IMD), Glutathione (GSH)	Remain from affinity purification procedures
9	Other Ions and Molecules	Chloride (Cl^-), Sodium (Na^+), Acetate (ACT)	Originate from buffers and crystallization salts

Table S3: List of unwanted ligands as used to create the ActivityDB database instance presented in this work.

HET code	Category
UNL	Dummies
DUM	Dummies
3CO	Ions
4MO	Ions
6MO	Ions
AG	Ions
AL	Ions
AR	Ions
AU	Ions
AU3	Ions
BA	Ions
BR	Ions
CA	Ions
CD	Ions
CE	Ions
CL	Ions
CMO	Ions
CO	Ions
CR	Ions
CS	Ions
CU	Ions

Continued on next page

HET code	Category
CU1	Ions
CYN	Ions
EU	Ions
EU3	Ions
F	Ions
FE	Ions
FE2	Ions
GA	Ions
GD	Ions
GD3	Ions
HDZ	Ions
HG	Ions
HO	Ions
HO3	Ions
IOD	Ions
IR3	Ions
K	Ions
KR	Ions
LA	Ions
LI	Ions
LU	Ions
MG	Ions
MN	Ions
NA	Ions

Continued on next page

HET code	Category
NI	Ions
PT	Ions
OS4	Ions
OXY	Ions
PB	Ions
PD	Ions
RB	Ions
PER	Ions
PR	Ions
SM	Ions
SR	Ions
TB	Ions
SX	Ions
TL	Ions
U1	Ions
W	Ions
XE	Ions
YB	Ions
YT3	Ions
ZN	Ions
VO4	Ions
PO3	Ions
DMS	Solvents
GOL	Solvents

Continued on next page

HET code	Category
GOL	Cryoprotectants
EDO	Cryoprotectants
MPD	Cryoprotectants
PGO	Cryoprotectants
SUC	Cryoprotectants
MLI	Cryoprotectants
TRE	Cryoprotectants
PEG	Cryoprotectants
IPA	Cryoprotectants
ACN	Cryoprotectants
EOH	Cryoprotectants
PEG	Precipitating_Agents
SO4	Precipitating_Agents
PO4	Precipitating_Agents
PEO	Precipitating_Agents
1PE	Precipitating_Agents
P33	Precipitating_Agents
P4G	Precipitating_Agents
P6G	Precipitating_Agents
P8K	Precipitating_Agents
2PE	Precipitating_Agents
3PE	Precipitating_Agents
4PE	Precipitating_Agents
PE3	Precipitating_Agents

Continued on next page

HET code	Category
PE4	Precipitating_Agents
PE5	Precipitating_Agents
PE6	Precipitating_Agents
PE7	Precipitating_Agents
PE8	Precipitating_Agents
PG4	Precipitating_Agents
PGE	Precipitating_Agents
ACT	Precipitating_Agents
CIT	Precipitating_Agents
TAR	Precipitating_Agents
MAL	Precipitating_Agents
MLI	Precipitating_Agents
MPD	Precipitating_Agents
EOH	Precipitating_Agents
IPA	Precipitating_Agents
ACN	Precipitating_Agents
SUC	Precipitating_Agents
AMS	Precipitating_Agents
FMT	Precipitating_Agents
TRS	Buffer_Molecules
HEZ	Buffer_Molecules
MES	Buffer_Molecules
BIS	Buffer_Molecules
IMD	Buffer_Molecules

Continued on next page

HET code	Category
CAC	Buffer_Molecules
CIT	Buffer_Molecules
PIP	Buffer_Molecules
BES	Buffer_Molecules
ADA	Buffer_Molecules
MOH	Buffer_Molecules
EPE	Buffer_Molecules
ACT	Buffer_Molecules
PO4	Buffer_Molecules
MLI	Buffer_Molecules
TAR	Buffer_Molecules
MAL	Buffer_Molecules
BOG	Detergents
LDA	Detergents
C8E	Detergents
DDM	Detergents
DM	Detergents
OG	Detergents
NG	Detergents
HTG	Detergents
LMT	Detergents
C12E8	Detergents
C12E9	Detergents
NTG	Detergents

Continued on next page

HET code	Category
OTG	Detergents
LDAO	Detergents
CHAPS	Detergents
CHAPSO	Detergents
FC12	Detergents
FC14	Detergents
DDN	Detergents
TX	Detergents
NP40	Detergents
B3C	Detergents
SDS	Detergents
CPS	Detergents
BNG	Detergents
UDM	Detergents
HDM	Detergents
TDM	Detergents
DEC	Detergents
HEX	Detergents
CYF	Detergents
CYH	Detergents
ZWT	Detergents
DTT	Reducing_Agents
BME	Reducing_Agents
GSH	Reducing_Agents

Continued on next page

HET code	Category
CYS	Reducing_Agents
THP	Reducing_Agents
BU3	Reducing_Agents
DTE	Reducing_Agents
HG	Heavy_Metal_Compounds
AU	Heavy_Metal_Compounds
PT	Heavy_Metal_Compounds
PB	Heavy_Metal_Compounds
UNX	Heavy_Metal_Compounds
AG	Heavy_Metal_Compounds
CD	Heavy_Metal_Compounds
IR3	Heavy_Metal_Compounds
OS4	Heavy_Metal_Compounds
RU	Heavy_Metal_Compounds
RH	Heavy_Metal_Compounds
EU	Heavy_Metal_Compounds
GD	Heavy_Metal_Compounds
TB	Heavy_Metal_Compounds
YB	Heavy_Metal_Compounds
SM	Heavy_Metal_Compounds
LU	Heavy_Metal_Compounds
HO	Heavy_Metal_Compounds
ER	Heavy_Metal_Compounds
DY	Heavy_Metal_Compounds

Continued on next page

HET code	Category
W	Heavy_Metal_Compounds
RE	Heavy_Metal_Compounds
MPD	Organic_Solvents_And_Additives
IPA	Organic_Solvents_And_Additives
EOH	Organic_Solvents_And_Additives
IPH	Organic_Solvents_And_Additives
DMS	Organic_Solvents_And_Additives
ACN	Organic_Solvents_And_Additives
MOH	Organic_Solvents_And_Additives
ACO	Organic_Solvents_And_Additives
THF	Organic_Solvents_And_Additives
DMF	Organic_Solvents_And_Additives
CHX	Organic_Solvents_And_Additives
HEX	Organic_Solvents_And_Additives
TOL	Organic_Solvents_And_Additives
BEN	Organic_Solvents_And_Additives
CHC	Organic_Solvents_And_Additives
DCM	Organic_Solvents_And_Additives
ETA	Organic_Solvents_And_Additives
BDN	Organic_Solvents_And_Additives
PDO	Organic_Solvents_And_Additives
BTO	Organic_Solvents_And_Additives
PTO	Organic_Solvents_And_Additives
DTO	Organic_Solvents_And_Additives

Continued on next page

HET code	Category
MTT	Organic_Solvents_And_Additives
DET	Organic_Solvents_And_Additives
PYR	Organic_Solvents_And_Additives
NMM	Organic_Solvents_And_Additives
TEA	Organic_Solvents_And_Additives
FOR	Organic_Solvents_And_Additives
ACE	Organic_Solvents_And_Additives
TFA	Organic_Solvents_And_Additives
HIS	Purification_Tags_And_Linkers
GST	Purification_Tags_And_Linkers
MBP	Purification_Tags_And_Linkers
NTA	Purification_Tags_And_Linkers
IDA	Purification_Tags_And_Linkers
STI	Purification_Tags_And_Linkers
CL	Other_Ions_And_Molecules
NA	Other_Ions_And_Molecules
K	Other_Ions_And_Molecules
CA	Other_Ions_And_Molecules
MG	Other_Ions_And_Molecules
BR	Other_Ions_And_Molecules
IOD	Other_Ions_And_Molecules
F	Other_Ions_And_Molecules
LI	Other_Ions_And_Molecules
RB	Other_Ions_And_Molecules

Continued on next page

HET code	Category
CS	Other_Ions_And_Molecules
SR	Other_Ions_And_Molecules
BA	Other_Ions_And_Molecules
ZN	Other_Ions_And_Molecules
MN	Other_Ions_And_Molecules
FE	Other_Ions_And_Molecules
FE2	Other_Ions_And_Molecules
FE3	Other_Ions_And_Molecules
CU	Other_Ions_And_Molecules
CU1	Other_Ions_And_Molecules
CU2	Other_Ions_And_Molecules
CO	Other_Ions_And_Molecules
NI	Other_Ions_And_Molecules
AL	Other_Ions_And_Molecules
MO	Other_Ions_And_Molecules
V	Other_Ions_And_Molecules
CR	Other_Ions_And_Molecules
SE	Other_Ions_And_Molecules
NH4	Other_Ions_And_Molecules
NO3	Other_Ions_And_Molecules
SO4	Other_Ions_And_Molecules
PO4	Other_Ions_And_Molecules
CO3	Other_Ions_And_Molecules
HCO3	Other_Ions_And_Molecules

Continued on next page

HET code	Category
ACY	Other_Ions_And_Molecules
FMT	Other_Ions_And_Molecules
OXL	Other_Ions_And_Molecules
SCN	Other_Ions_And_Molecules
AZI	Other_Ions_And_Molecules
OH	Other_Ions_And_Molecules
O	Other_Ions_And_Molecules
O2	Other_Ions_And_Molecules
CIT	Other_Ions_And_Molecules
MLI	Other_Ions_And_Molecules
SUC	Other_Ions_And_Molecules

4 HET code variations

Table S4: Distribution of HET Codes by Number of Variants (variants = distinct standard InChI-Keys per HET code)

Number of variants	Number of HET codes	Percentage (%)
1	31 370	91.530
2	1 946	5.678
3	438	1.278
4	157	0.458
5	76	0.222
6	54	0.158

Continued on next page

Continued from previous page

Number of variants	Number of HET codes	Percentage (%)
7	41	0.120
8	34	0.099
9	20	0.058
10	14	0.041
11	12	0.035
12	11	0.032
13	10	0.029
14	11	0.032
15	6	0.018
16	5	0.015
17	7	0.020
18	2	0.006
19	4	0.012
20	3	0.009
21	1	0.003
22	2	0.006
23	3	0.009
24	4	0.012
25	1	0.003
26	1	0.003
28	1	0.003
29	1	0.003
30	2	0.006

Continued on next page

Continued from previous page

Number of variants	Number of HET codes	Percentage (%)
31	2	0.006
33	2	0.006
35	2	0.006
37	2	0.006
39	1	0.003
44	1	0.003
46	1	0.003
50	1	0.003
51	1	0.003
52	1	0.003
56	2	0.006
57	2	0.006
58	2	0.006
62	1	0.003
68	1	0.003
71	1	0.003
75	1	0.003
76	1	0.003
79	1	0.003
91	2	0.006
93	1	0.003
102	1	0.003
104	1	0.003

Continued on next page

Continued from previous page

Number of variants	Number of HET codes	Percentage (%)
124	1	0.003
210	1	0.003
219	1	0.003
299	1	0.003
377	1	0.003

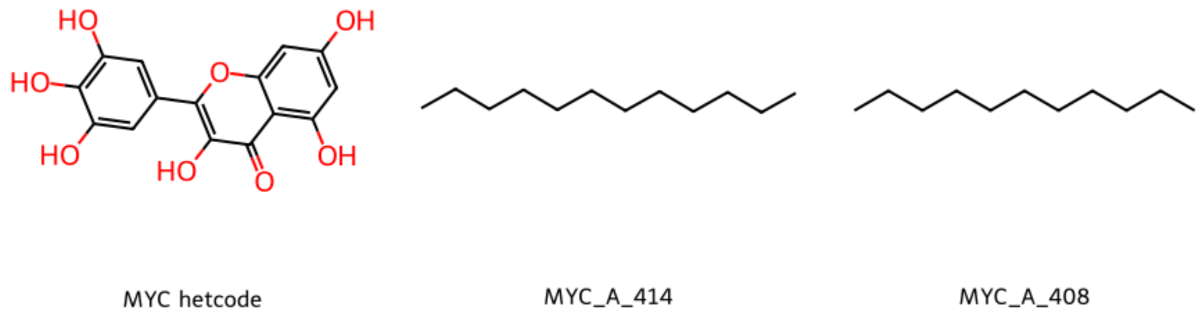

Figure S1: Example of the MYC case in `5HXC`[S1,S2] where the original ligand is modeled in multiple instances as simple alkane.

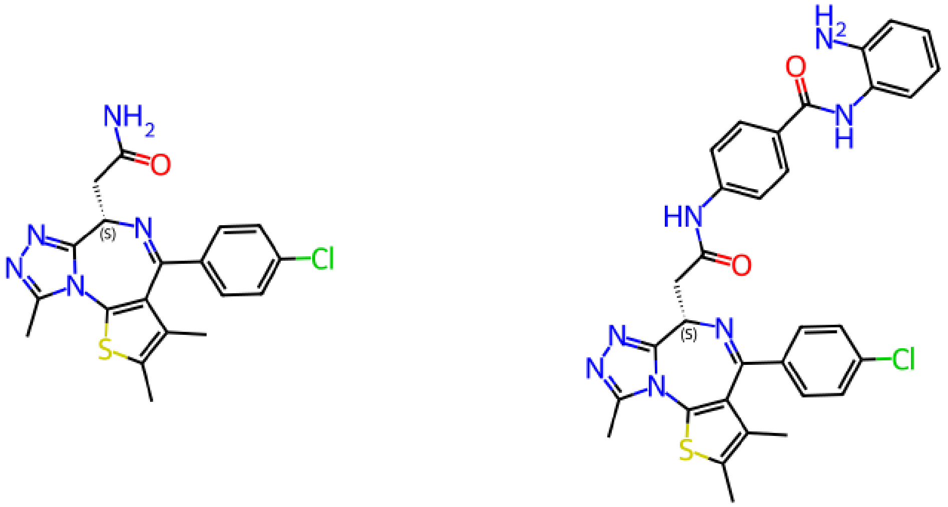

P8T built from 6YQN and CHEMBL4303293

Hetcode P8T and CHEMBL5197137

(a)

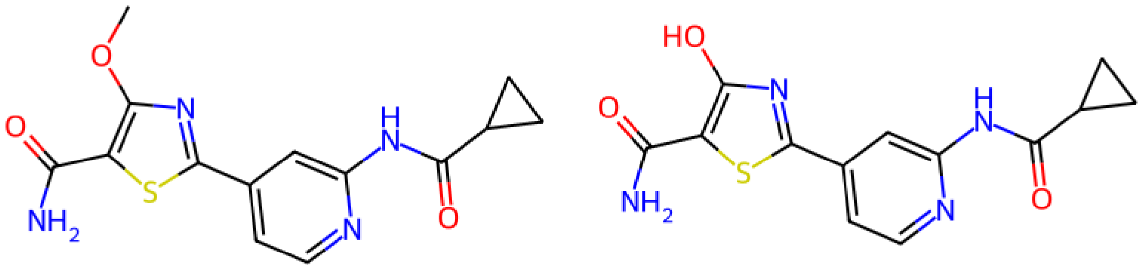

2WE hetcode

2WE in 4PTC

(b)

Figure S2: Example of incompletely modelled molecules. The P8T case in 6YQN[S3,S4] is displayed in a) and the 2WE case in 4PTC[S5,S6] in b). This may result in an alternative ChEMBL molecules being linked by ActivityFinder and BioChemGraph.

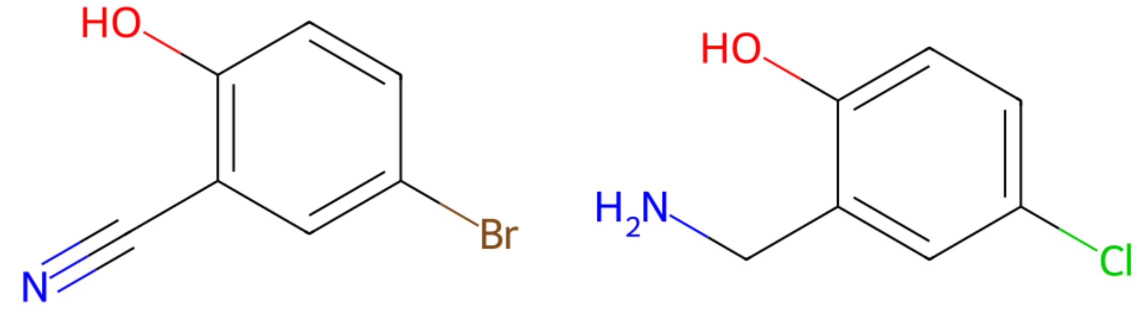

UUJ CHEMBL33719

(a)

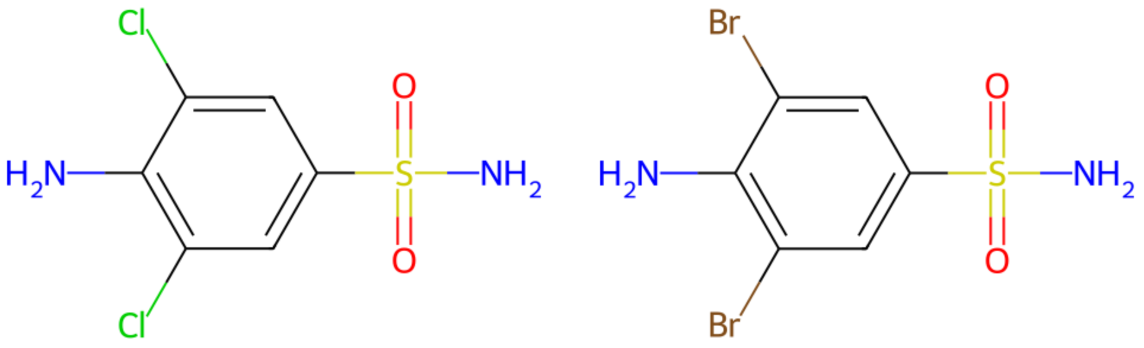

SDA CHEMBL306195

(b)

Figure S3: Exemplary links at molecule matching level 1 (a) and level 2 (b). For links in level 2 both the connection and hydrogen layer of the `InChI` need to be identical while for level 1 the hydrogen layer and therefore protonation is allowed to differ.

5 Mutations in `Variant` Sequences

Table S5: Distribution of Mutation Counts in ChEMBL Variant Sequences

**Number of mutations**	**Number of sequences**	**Percentage (%)**
1	2 170	88.825
2	201	8.228
3	46	1.883
4	12	0.491
5	3	0.123
6	4	0.164
7	1	0.041
8	1	0.041
9	1	0.041
10	1	0.041
13	1	0.041
17	1	0.041
20	1	0.041

6 Sequence Matching Results

Table S6: Percent identity interval-binned raw counts of unique PDB-ChEMBL sequence pairs used to generate the stacked bars in Figure 8. Counts are shown separately for the Component and Variant sides, as well as for each PDB sequence type. The bottom "Total" row is the sum of the interval counts (i.e., the bar annotations in the absolute panels). Unique sequence counts: `ATOM` = 99 961; `SEQRES` = 43 867; `SEQADV` = 21 183.

% identity	Component			Variant		
	ATOM	SEQRES	SEQADV	ATOM	SEQRES	SEQADV
[100]	9 786	7 354	5 857	3 185	1 769	386
[95,100)	36 873	16 120	6 337	48 028	27 860	3 032
[90,95)	28 442	6 994	3 128	38 972	9 487	376
[85,90)	20 532	6 485	3 175	21 357	7 979	240
[80,85)	14 588	5 354	2 483	9 946	3 956	261
Total	110 221	42 307	20 980	121 488	51 051	4 295

Table S7: Percent identity interval composition (percent of RAW unique PDB-ChEMBL sequence pairs within each sequence type) for sequence pairs with percent identity $\geq 80\,\%$. These values produce the two normalized panels of Figure 8. Percentages are derived from the raw interval counts (see Table S6). For example, `COMPONENT-ATOM`: $[95, 100) = 36\,873/110\,221 = 33.45\,\%$. Minor rounding ensures each column sums to $100.00\,\%$.

% Identity	Component (%)			Variant (%)		
	ATOM	SEQRES	SEQADV	ATOM	SEQRES	SEQADV
[100]	8.88	17.38	27.92	2.62	3.47	8.99
[95,100)	33.45	38.10	30.20	39.53	54.57	70.59
[90,95)	25.80	16.53	14.91	32.08	18.58	8.75
[85,90)	18.63	15.33	15.13	17.58	15.63	5.59
[80,85)	13.24	12.66	11.84	8.19	7.75	6.08
Sum	100.00	100.00	100.00	100.00	100.00	100.00

7 Activity Qualified Ligand Matching Results

Table S8: Activity-qualified distribution of distinct ligand mapping pairs (pdblid, clid) across maximum BLAST sequence identity intervals. Each pair appears exactly once, assigned via its maximum observed percent identity ($\geq$ 80 %) among activity-supported sequence mappings; structural confidence (smmclid) is the highest structural matching level observed. Percent columns: within-interval and global.

Identity Interval	smmclid	Pairs	Interval Total	% Interval	% Global
[100]	1	2 975	17 173	17.32	13.915
[100]	2	1 067	17 173	6.21	4.991
[100]	3	3 606	17 173	21.0	16.866
[100]	4	591	17 173	3.44	2.764
[100]	5	8 934	17 173	52.02	41.787
[95, 100)	1	491	2 387	20.57	2.297
[95, 100)	2	155	2 387	6.49	0.725
[95, 100)	3	605	2 387	25.35	2.83
[95, 100)	4	73	2 387	3.06	0.341
[95, 100)	5	1 063	2 387	44.53	4.972
[90, 95)	1	215	902	23.84	1.006
[90, 95)	2	44	902	4.88	0.206
[90, 95)	3	225	902	24.94	1.052
[90, 95)	4	36	902	3.99	0.168
[90, 95)	5	382	902	42.35	1.787
[85, 90)	1	159	523	30.4	0.744
[85, 90)	2	41	523	7.84	0.192
[85, 90)	3	120	523	22.94	0.561
[85, 90)	4	22	523	4.21	0.103
[85, 90)	5	181	523	34.61	0.847
[80, 85)	1	141	395	35.7	0.659
[80, 85)	2	8	395	2.03	0.037
[80, 85)	3	92	395	23.29	0.43
[80, 85)	4	7	395	1.77	0.033
[80, 85)	5	147	395	37.22	0.688

8 Detailed Comparison to BioChemGraph

Excellent resources exist in the public domain trying to make it possible to link identical proteins or identical molecules. For the protein side of things, it is possible to link proteins in different databases using UniProt IDs. Moreover, one can link identical molecules using canonical USMILES (if the same source has calculated them) or the `InChI` (or `InChIKey`), which has been developed explicitly for this. Resources like BioChemGraph or Papyrus utilize both UniProt IDs and `InChIKey` to link identical molecules and protein sequences between, in the case of BioChemGraph, the CSD, PDB, and ChEMBL. While this approach is not extendible to - for example - proprietary datasets, both BioChemGraph and ActivityFinder create a link between PDB and ChEMBL. This allows us to easily compare the results of an identifier-based approach and our more complex method and discuss their advantages and disadvantages.

8.1 Comparison to identifier-based small molecule matching

As with all topics regarding linking structural and bioactivity data, linking small molecules from different databases initially seems easy, but is complex in detail. When comparing the linking BioChemGraph does using the `InChIKey` and our linking based on the small-molecules built from each PDB structures, we find 9 913 mappings of hetcodes to ChEMBL ligands from the BioChemGraph and 57 724 mappings of hetcodes to ChEMBL ligands with ActivityFinder out of which 48 602 are not contained in BioChemGraph (see Figure S4. This size difference is due to multiple reasons. The first and biggest is that to test our method only on linking small molecules, we extract all potential links without considering only those with matching bioactivities, as this would introduce the sequence matching side of things. Second, as we employ multiple matching levels, one PDB ligand can match multiple ChEMBL molecules, and various representations can exist for the same hetcode if it is modeled differently. It is not unusual for molecules with identical hetcodes to be modeled

differently in multiple or even the same PDB structure. This can happen because the ligand is only partially resolved in the electron density and is rightfully only partially built. Still, sometimes the differences can seem so stark that they are probably erroneous. One example is the ligand MYC in `5HXC`,[S7,S8] where the relatively complex ligand is modeled as multiple alkanes.

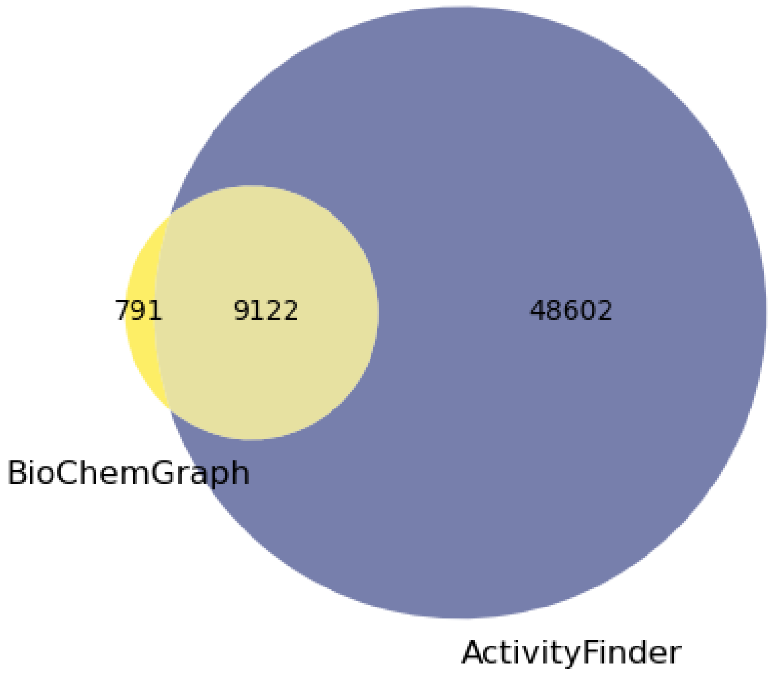

Figure S4: Visualisation on the overlap between hetcode-ChEMBL ligand combinations found by the BioChemGraph and ActivityFinder. Every blue combination was found only by ActivityFinder, dark yellow only by BioChemGraph, and light yellow by both approaches. Combinations in BioChemGraph are all found with at least one activity, while the combinations of ActivityFinder are solely molecule matches.

8.1.1 Identifier-based matchings not reproduced

Two reasons were found for the 791 cases in which only BioChemGraph finds a link, but ActivityFinder does not find the same link. The first reason is that ActivityFinder builds the molecule differently from the hetcode because it is modeled differently in the PDB file. This comes in multiple cases, the first being that ActivityFinder also finds a link to the ChEMBL for this hetcode but only to a different ChEMBL ligand, which happens in 18 cases. One example is the ligand 2WE, which is linked to CHEMBL3410089 by BioChemGraph but with matching `InChIKey` to CHEMBL4787873 by ActivityFinder, missing a methyl group. Investigating the only structure with the hetcode, PDB `4PTC`,[S5,S6] the methyl group, although present in the hetcode, is not modeled in the structure. Another example of this is the ligand 4KB, which is linked to CHEMBL3613776 by BioChemGraph but to CHEMBL3613780 by ActivityFinder with matching `InChIKeys`, missing two methoxy groups. The second case is that ActivityFinder also does not find a matching ligand in the ChEMBL. An example is the ligand 17J, which is linked to CHEMBL513553 with BioChemGraph. It is only modeled in `4HBP`[S9,S10] and is covalently bound to the protein, with a part of the structure not modeled, resulting in the difference. The second reason is readin issues. As ActivityFinder only processes crystal structures, some ligands like 05X in the electron microscopy structure `7EXD`[S11,S12] were not added to the `ActivityDB` upon construction.

8.1.2 Identifier-based matchings reproduced at a different matching level

When investigating cases where both approaches link the same molecules, there are 845 duplicate cases in which we build the same hetcode in multiple ways. Therefore, there are 9 967 cases where both approaches link the same hetcode and ChEMBL molecule. Of these cases, only in 8 238 of 9 967 (82.65 %), we create the link with an identical `InChIKey`. The rest is linked with "canonical smiles with chiral information" (426/4.27 %), "canonical smiles without chiral information" (476/4.78 %), "clipped `InChI` connection and hydrogen layer (3/0.03 %), or "clipped `InChI` connection layer" (8248.27 %).

Again, these are cases where the difference is due to different ligands being built from the PDB file. One severe example is ligand CQD in PDB 4LMT, where despite a flat aromatic bicyclic compound described in the hetcode, the modeled structure is not flat (Figure S6a). Another example is the ligand 5LO linked to CHEMBL3781132, where ActivityFinder constructs an additional double bond in the cyclopentane ring, altering the molecule so that it is only matched when going down to the lowest matching level (Figure S6b). For the ligand BRH (Figure S6c), both approaches link to the racemic ligand CHEMBL73698. However, we do this with USMILES without chiral information, as the ligand modeled in the PDB is not racemic. The string representations of both PDB and ChEMBL are racemic; therefore, the `InChI` perfectly match, even though the name in the PDB, the specific chiral form, is specified. The ligand MYC in the `2063`[S13,S14] is one example where the low matching levels become important (see Figure S5). It is linked by BioChemGraph to

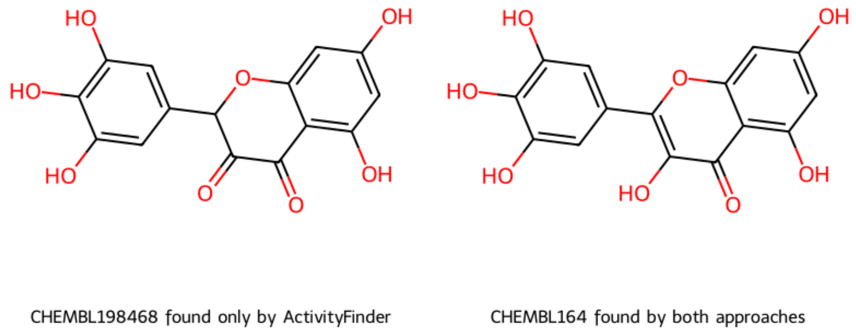

Figure S5: Linked ChEMBL molecules for hetcode MYC in `2063`[S13,S14] where both approaches link to the same molecule, but ActivityFinder finds an alternative ChEMBL molecule with a different tautomerisation.

CHEMBL164, which ActivityFinder also links with an `InChIKey` match. In addition, ActivityFinder links CHEMBL2048508, CHEMBL3348861, CHEMBL5426064, CHEMBL5403463, and CHEMBL198468 all with the lowest molecule matching level, the clipped connection layer of the `InChI`. While the first four instances are slightly different ligands, even with different molecular weights, the latter is a tautomeric form of the first ligand (Figure S6d).

While these matches are challenging to incorporate into automatically constructed datasets, they add valuable information for scientists investigating these entries manually. This is especially true for cases where no `InChIKey` match exists.

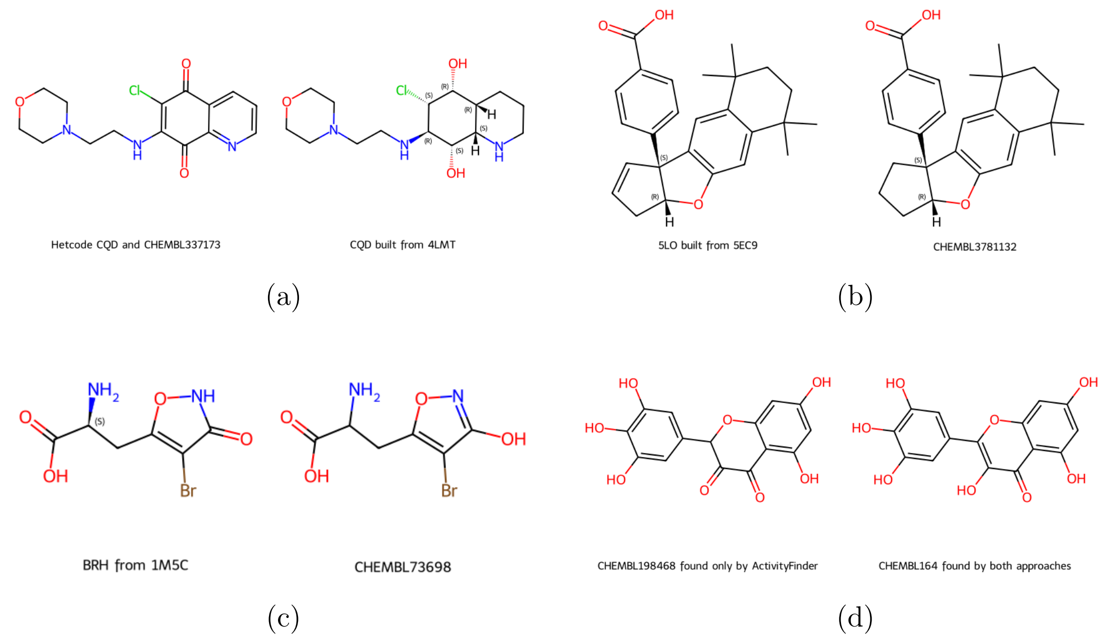

Figure S6: Cases in which both BioChemGraph and ActivityFinder link the same hetcode but either with different matching levels or with additional identical molecules.

8.1.3 Matchings only found by ActivityFinder

Starting with the 48 602 cases where only ActivityFinder finds a mapping, there are again some duplicate entries increasing the number of mappings to 62 886. In 10 401/16.54 % one with an `InChIKey` match, highlighting how frequently identical molecules are found with a target not matching by UniProt ID. One example of a link that only ActivityFinder finds, even though the `InChIKey` of both ChEMBL and PDB representation match, is LLT to CHEMBL374731, which is an approved drug called telbivudine used to treat hepatitis B and is present in `3HP1`[S15,S16] and `3QEO`[S17,S18] (see Figure S7d).

One further problem with small-molecule matching is recognizing `InChI` or `USMILES` that are different but describe practically identical molecules for the drug design community. We found two cases of this, molecules being described as multiple disconnected parts in

the ChEMBL or molecules with specified isotopes and therefore rightfully not matching their counterparts by trivial `InChIKey` comparison. Because ActivityFinder only takes the largest component of mixtures when processing small molecules from ChEMBL, it can additionally link the hetcode H4B with CHEMBL1201775, while BioChemGraph only links it to CHEMBL1201774 (Figure S7a). The difference between the two entries is that salts are present, but the main component—the approved drug sapropterin—remains unchanged. This could be solved by ChEMBL functionality of alternative compound forms but is currently not automatically done by BioChemGraph. Another example of this for a ligand that only ActivityFinder finds is the link between 3BT and CHEMBL1527769 (Figure S7b). Removing isotopes during small-molecule processing allows ActivityFinder to also link molecules like the hetcode MC and CHEMBL3350473, despite their difference in one isotope (Figure S7c).

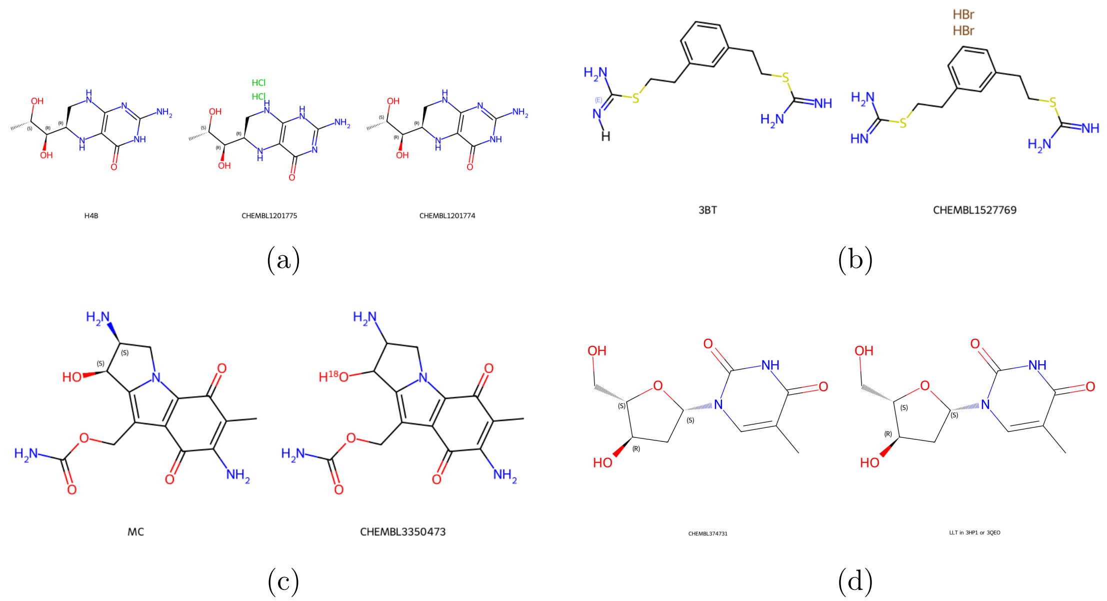

Figure S7: Cases in which only ActivityFinder finds the link between the hetcode and ChEMBL molecule.

8.2 Comparison to identifier-based sequence matching

The second big part of matching activity and structural data is protein matching. One potential problem is that our sequence-based approach does not reproduce the UniProt match. As ActivityFinder also has a target mode where only the sequence is considered, this allows the investigation of all found PDB-ChEMBL target combinations that ActivityFinder could construct. As is immediately apparent in the Venn Diagram in Figure S8, we find many more potential PDB-ChEMBL target combinations using the target mode than BioChemGraph. It is important to note that this is expected because we do not restrict our search to targets with active ligands and consider everything above 80% sequence identity.

However, we do not find the identical PDB-target combination for 3 137 of the 18 767 (ca. 16.72%) combinations of BioChemGraph. Further investigating these cases, only 175 of them (ca. 6.8%) are without PDB read-in issues. The read-in issues are primarily due to filtering any experiment type apart from crystal structures. Apart from that, it happens in 422 cases that NAOMI cannot construct any ligand from the PDB files. These 422 cases seem to be dominated by covalently bound ligands. Additionally, 547 cases that are not part of our PDB mirror. When investigating the remaining 175 missing links, it became apparent that 50 are due to a sequence identity below 80 %. One, albeit rare, problem with the approach of BioChemGraph is that sometimes UniProt identifiers are matched to small parts of a structure in the PDB. One such example is the `7YMJ`[S19,S20] where the UniProt identifiers P35348 and P41145 are present, but the latter only for a small part of the structure that is not directly interacting with the found Ligand tamsulosin. The best possible sequence identity between `7YMJ`[S19,S20] and P41145 is 32.4%, highlighting that any activities correlated should not be trusted. Other examples are `5SYO`[S21] or `4ODR`.[S22,S23] However, this problem is infrequent, as the best-calculated sequence identities for the `SEQRES` and UniProt sequences in all BioChemGraph links in Figure 11 showcase. Lastly, there are 125 cases, which is 0.66 % of the links BioChemGraph creates, for which we are unaware why our sequence-

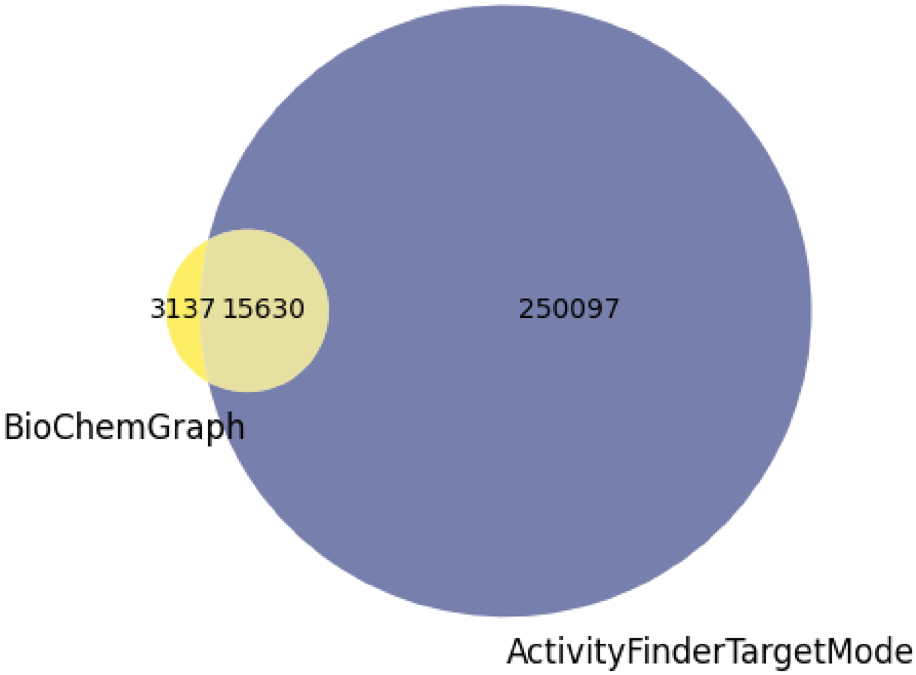

Figure S8: Visualization of the overlap between pdb-target combinations found by the BioChemGraph and ActivityFinder in the target mode. A combination is defined as a combination of a PDB, ChEMBL target. Every blue combination was found only by ActivityFinder, dark yellow only by BioChemGraph, and light yellow by both approaches. Combinations in BioChemGraph are all found with at least one activity. In contrast, the combinations of ActivityFinder are solely sequence matches.

based approach does not reproduce the exact ChEMBL target matching.

8.3 Combinations unique to BioChemGraph

The reasons why ActivityFinder does not reproduce 4 562 combinations can be split into four categories. The first is readin problems (3 110 / 68.17 %) with the given PDB structure, which is again partially due to structures not in our PDB mirror (557 / 17.91 %) and structures that are outside the scope of ActivityFinder, like structures determined by cryo-EM (2 065 / 66.4 %). 428 (13.76 %) structures cannot be correctly read in for other reasons, like the PDB structure not having valid ligands. The second issue is the differences between ChEMBL and `ActivityDB` (456 / 9.99 %). These consist of 42 cases in which both the ChEMBL molecule and the activity is not in the `ActivityDB` and of 446 cases where only the activity is missing. The third issue is PDB-target connections (140 / 3.01 %) from the BioChemGraph not reproduced by ActivityFinder. For 50 (1.1 %) of these, the best possible sequence identity calculated between PDB and UniProt sequence is below 80 %. The last issue is that the ligand is not reproduced by ActivityFinder (856 / 18.76 %), which can again be split into two cases. The first one was that the PDB file's ligand correlated with a different ChEMBL ligand than in the BioChemGraph (139 / 3.04 %). In the second case, the het code in the PDB structure is correlated to the identical ChEMBL ligand but not successfully processed for a different reason (717 / 15.72 %).

8.4 Sequence matching settings between ActivityFinder and BioChemGraph

The best possible sequence identities were calculated for all `SEQRES` sequences found in the PDB file compared to the UniProt sequence annotated by BioChemGraph. Sequence Identity was calculated with BioPython[S24] using the Pairwise Aligner with the global alignment option. To best resemble the matching constructed by ActivityFinder, a match score of

2, mismatch score of −1, open gap score of −10 and extend gap score of −0.5 was used. Sequence identity was then calculated by counting all identical characters excluding gaps and normalising on sequence length.

References

(S1) Liao, J.; Jiang, Y.; Faraldo-Gomez, J. Structural mechanisms of extracellular ion exchange and induced binding-site occlusion in the sodium-calcium exchanger NCX_Mj soaked with 20 mM Na+ and zero Ca2+: 5hxc. https://www.wwpdb.org/pdb?id=pdb_00005hxc, Institution: Worldwide Protein Data Bank.

(S2) Liao, J.; Marinelli, F.; Lee, C.; Huang, Y.; Faraldo-Gómez, J. D.; Jiang, Y. Mechanism of extracellular ion exchange and binding-site occlusion in a sodium/calcium exchanger. *23*, 590–599.

(S3) Joerger, A.; Balourdas, D.; Weiser, T.; Chatterjee, D.; Knapp, S.; Structural Genomics Consortium (SGC) Crystal structure of the first bromodomain of human BRD4 in complex with the dual inhibitor TW9: 6yqn. https://www.wwpdb.org/pdb?id=pdb_00006yqn, Institution: Worldwide Protein Data Bank.

(S4) Zhang, X.; Zegar, T.; Weiser, T.; Hamdan, F. H.; Berger, B.-T.; Lucas, R.; Balourdas, D.-I.; Ladigan, S.; Cheung, P. F.; Liffers, S.-T.; others Characterization of a dual BET/HDAC inhibitor for treatment of pancreatic ductal adenocarcinoma. *International Journal of Cancer* **2020**, *147*, 2847–2861.

(S5) Lewis, H.; Sivaprakasam, P.; Kish, K.; Pokross, M.; Dubowchik, G. Structure of a carboxamide compound (3) (2-2-[(CYCLOPROPYLCARBONYL)AMINO]PYRIDIN-4-YL-4-OXO-4H-1LAMBDA 4 ,3-THIAZOLE-5-CARBOXAMIDE) to GSK3b. https://www.wwpdb.org/pdb?id=pdb_00004ptc, Institution: Worldwide Protein Data Bank.

(S6) Sivaprakasam, P.; Han, X.; Civiello, R. L.; Jacutin-Porte, S.; Kish, K.; Pokross, M.; Lewis, H. A.; Ahmed, N.; Szapiel, N.; Newitt, J. A.; Baldwin, E. T.; Xiao, H.; Krause, C. M.; Park, H.; Nophsker, M.; Lippy, J. S.; Burton, C. R.; Langley, D. R.; Macor, J. E.; Dubowchik, G. M. Discovery of new acylaminopyridines as GSK-3 inhibitors by a structure guided in-depth exploration of chemical space around a pyrrolopyridinone core. *25*, 1856–1863.

(S7) Liao, J.; Jiang, Y.; Faraldo-Gomez, J. Structural mechanisms of extracellular ion exchange and induced binding-site occlusion in the sodium-calcium exchanger NCX_Mj soaked with 20 mM Na+ and zero Ca2+: 5hxc. https://www.wwpdb.org/pdb?id=pdb_00005hxc, Institution: Worldwide Protein Data Bank.

(S8) Liao, J.; Marinelli, F.; Lee, C.; Huang, Y.; Faraldo-Gómez, J. D.; Jiang, Y. Mechanism of extracellular ion exchange and binding-site occlusion in a sodium/calcium exchanger. *23*, 590–599.

(S9) Behnke, C.; Skene, R. Crystal Structure of FAAH in complex with inhibitor: 4hbp. https://www.wwpdb.org/pdb?id=pdb_00004hbp, Institution: Worldwide Protein Data Bank.

(S10) Kono, M.; Matsumoto, T.; Kawamura, T.; Nishimura, A.; Kiyota, Y.; Oki, H.; Miyazaki, J.; Igaki, S.; Behnke, C. A.; Shimojo, M.; Kori, M. Synthesis, SAR study, and biological evaluation of a series of piperazine ureas as fatty acid amide hydrolase (FAAH) inhibitors. *21*, 28–41.

(S11) Huang, S.; Xu, P.; Jiang, Y.; Xu, H. Lasmiditan-bound serotonin 1F (5-HT1F) receptor-Gi protein complex: 7exd. https://www.wwpdb.org/pdb?id=pdb_00007exd, Institution: Worldwide Protein Data Bank.

(S12) Huang, S.; Xu, P.; Tan, Y.; You, C.; Zhang, Y.; Jiang, Y.; Xu, H. E. Structural basis

for recognition of anti-migraine drug lasmiditan by the serotonin receptor 5-HT1F–G protein complex. *31*, 1036–1038.

(S13) Holder, S.; Zemskova, M.; Zhang, C.; Tabrizizad, M.; Bremer, R.; Neidigh, J.; Lilly, M. Crystal structure of Pim1 with Myricetin: 2o63. https://www.wwpdb.org/pdb?id=pdb_00002o63, Institution: Worldwide Protein Data Bank.

(S14) Holder, S.; Zemskova, M.; Zhang, C.; Tabrizizad, M.; Bremer, R.; Neidigh, J. W.; Lilly, M. B. Characterization of a potent and selective small-molecule inhibitor of the PIM1 kinase. *Molecular cancer therapeutics* **2007**, *6*, 163–172.

(S15) Hazra, S.; Lavie, A. Crystal structure of human dCK R104M/D133A in complex with L-dT and ADP: 3hp1. https://www.wwpdb.org/pdb?id=pdb_00003hp1, Institution: Worldwide Protein Data Bank.

(S16) Hazra, S.; Sabini, E.; Ort, S.; Konrad, M.; Lavie, A. Extending Thymidine Kinase Activity to the Catalytic Repertoire of Human Deoxycytidine Kinase. *48*, 1256–1263.

(S17) Lavie, A.; Hazra, S. S74E-R104M-D133A dCK variant in complex with L-deoxythymidine and UDP: 3qeo. https://www.wwpdb.org/pdb?id=pdb_00003qeo, Institution: Worldwide Protein Data Bank.

(S18) Hazra, S.; Szewczak, A.; Ort, S.; Konrad, M.; Lavie, A. Post-Translational Phosphorylation of Serine 74 of Human Deoxycytidine Kinase Favors the Enzyme Adopting the Open Conformation Making It Competent for Nucleoside Binding and Release. *50*, 2870–2880.

(S19) Toyoda, Y.; Zhu, A.; Yan, C.; Kobilka, B.; Liu, X. Cryo-EM structure of alpha1AAR-Nb6 complex bound to tamsulosin: 7ymj. https://www.wwpdb.org/pdb?id=pdb_00007ymj, Institution: Worldwide Protein Data Bank.

(S20) Toyoda, Y.; Zhu, A.; Kong, F.; Shan, S.; Zhao, J.; Wang, N.; Sun, X.; Zhang, L.; Yan, C.; Kobilka, B. K.; Liu, X. Structural basis of alpha1A-adrenergic receptor activation and recognition by an extracellular nanobody. *14*, 3655.

(S21) Bobango, J.; Wu, J.; Talley, I.; Talley, T. Crystal structure of a chimeric acetylcholine binding protein from Aplysia californica (Ac-AChBP) containing loop C from the human alpha 3 nicotinic acetylcholine receptor in complex with Cytisine: 5syo. `https://www.wwpdb.org/pdb?id=pdb_00005syo`, Institution: Worldwide Protein Data Bank.

(S22) Quistgaard, E.; Low, C.; Nordlund, P. Structure of SlyD delta-IF from Thermus thermophilus in complex with FK506. `https://www.wwpdb.org/pdb?id=pdb_00004odr`, Institution: Worldwide Protein Data Bank.

(S23) Quistgaard, E. M.; Weininger, U.; Ural-Blimke, Y.; Modig, K.; Nordlund, P.; Akke, M.; Löw, C. Molecular insights into substrate recognition and catalytic mechanism of the chaperone and FKBP peptidyl-prolyl isomerase SlyD. *14*, 82.

(S24) Cock, P. J.; Antao, T.; Chang, J. T.; Chapman, B. A.; Cox, C. J.; Dalke, A.; Friedberg, I.; Hamelryck, T.; Kauff, F.; Wilczynski, B.; others Biopython: freely available Python tools for computational molecular biology and bioinformatics. *Bioinformatics* **2009**, *25*, 1422.
